# Merkel Cell Polyomavirus DNA Replication Induces Senescence in Human Dermal Fibroblasts in a Kap1/Trim28-Dependent Manner

**DOI:** 10.1128/mBio.00142-20

**Published:** 2020-03-10

**Authors:** Svenja Siebels, Manja Czech-Sioli, Michael Spohn, Claudia Schmidt, Juliane Theiss, Daniela Indenbirken, Thomas Günther, Adam Grundhoff, Nicole Fischer

**Affiliations:** a Institute for Medical Microbiology, Virology and Hygiene, University Medical Center Hamburg-Eppendorf, Hamburg, Germany; b Heinrich Pette Institute, Leibniz Institute for Experimental Virology, Hamburg, Germany; University of Michigan—Ann Arbor

**Keywords:** Kap1/Trim28, Merkel cell polyomavirus, senescence, viral DNA replication, viral restriction factor

## Abstract

Merkel cell polyomavirus (MCPyV) is the only polyomavirus known to be associated with tumorigenesis in humans. Similarly to other polyomaviruses, MCPyV expresses a large tumor antigen (LT-Ag) that, together with a small tumor antigen (sT-Ag), contributes to cellular transformation and that is of critical importance for the initiation of the viral DNA replication. Understanding the cellular protein network regulated by MCPyV early proteins will significantly contribute to our understanding of the natural MCPyV life cycle as well as of the mechanisms by which the virus contributes to cellular transformation. We here describe KRAB-associated protein 1 (Kap1), a chromatin remodeling factor involved in cotranscriptional regulation, as a novel protein interaction partner of MCPyV T antigens sT and LT. Kap1 knockout results in a significant increase in the level of viral DNA replication that is highly suggestive of Kap1 being an important host restriction factor during MCPyV infection. Differently from other DNA viruses, MCPyV gene expression is unaffected in the absence of Kap1 and Kap1 does not associate with the viral genome. Instead, we show that in primary normal human dermal fibroblast (nHDF) cells, MCPyV DNA replication, but not T antigen expression alone, induces ataxia telangiectasia mutated (ATM) kinase-dependent Kap1 S824 phosphorylation, a mechanism that typically facilitates repair of double-strand breaks in heterochromatin by arresting the cells in G_2_. We show that MCPyV-induced inhibition of cell proliferation is mainly conferred by residues within the origin binding domain and thereby by viral DNA replication. Our data suggest that phosphorylation of Kap1 and subsequent Kap1-dependent G_2_ arrest/senescence represent host defense mechanisms against MCPyV replication in nHDF cells.

## Abstract (precis)

We here describe Kap1 as a restriction factor in MCPyV infection. We report a novel, indirect mechanism by which Kap1 affects MCPyV replication. In contrast with from other DNA viruses, Kap1 does not associate with the viral genome in MCPyV infection and has no impact on viral gene expression. In MCPyV-infected nHDF cells, Kap1 phosphorylation (pKap1 S824) accumulates because of genomic stress mainly induced by viral DNA replication. In contrast, ectopic expression of LT or LT MCPyV mutants, previously shown to be important for induction of genotoxic stress, does not result in a similar extent of pKap1 accumulation. We show that cells actively replicating MCPyV accumulate pKap1 (in a manner dependent on the presence of ATM) and display a senescence phenotype reflected by G_2_ arrest. These results are supported by transcriptome analyses showing that LT antigen, in a manner dependent on the presence of Kap1, induces expression of secreted factors, which is known as the senescence-associated secretory phenotype (SASP).

## INTRODUCTION

Merkel cell polyomavirus (MCPyV) is the causative agent of the majority (>60%) of Merkel cell carcinomas (MCCs), which represent a rare but highly aggressive form of skin cancer in immunosuppressed patients (1–3). The virus has been classified as a human-pathogenic tumor virus due to its causative role in tumor initiation (1, 2, 4–9).

MCPyV codes for the viral tumor antigens large tumor antigen (LT-Ag) and small tumor antigen (sT-Ag), which are responsible for viral DNA replication but furthermore play important roles in cell transformation and tumor maintenance (6, 10, 11). Although the virus was discovered more than 10 years ago, due to a lack of suitable *in vitro* and *in vivo* models, many aspects of the viral life cycle and of the viral mechanisms in MCC onset and progression are still unknown (3). Consequently, knowledge of the cellular proteins that positively or negatively affect the viral life cycle is very limited. Furthermore, the precise cell types that serve as the primary and/or persistent reservoirs of infection, as well as those that give rise to MCC, remain unknown. Currently, the MCPyV life cycle can be investigated in semipermissive replication systems (12–16) that permit viral DNA replication and limited particle production but not robust serial transmission. Recently, primary human dermal fibroblasts have been shown to support progeny production after *in vitro* and *ex vivo* infection with MCPyV (14).

Thus far, only a few MCPyV T-Ag interaction partners which affect viral replication have been described. In addition to the well-studied LT interaction partner retinoblastoma protein (Rb) (17–19), these include Vam6p, a protein involved in lysosome clustering (20), as well as chromatin (Ch)-binding bromodomain protein 4 (Brd4), which directly binds to LT and positively regulates MCPyV DNA replication (21). In a study designed to identify cellular binding partners of MCPyV early gene products by mass spectrometry, we recently identified KRAB-associated protein 1 (Kap1)/Trim28 as an additional LT-binding protein (22).

Kap1 is a multifunctional protein involved in chromatin remodeling, cotranscriptional repression, cell cycle regulation, and oncogene-induced senescence (23, 24). Kap1 was originally described as a retroviral restriction factor (25–27) but was also found to negatively affect transcription of different DNA viruses (28–33). The protein contains an RBCC domain that mediates interactions with transcription factors of the KRAB zinc finger family (KRAB-ZNFs), a TRIM-specific (TSS) domain, a heterochromatin protein (HP1)-binding domain, and a plant homeodomain (PHD), namely, the BROMO domain, with the latter two being particularly important for the protein’s chromatin remodeling function (23, 24). The C terminus of Kap1, which includes the TSS domain and the BROMO PHD, can be extensively modified by different posttranslational modifications, thereby regulating Kap1 function. SUMOylation of Kap1 facilitates transcriptional repression via histone deacetylation and trimethylation at lysine 9 of histone 3 (H3K9) by CHD3 and SETDB1. SUMOylation-dependent repression is counteracted either by de-SUMOylation through SENP1 or by phosphorylation of Kap1 on serine 824 (S824). Phosphorylation of Kap1 S824 is a key component of the DNA damage response pathway. Upon Kap1 S824 phosphorylation, heterochromatin is remodeled, thus rendering the DNA accessible to proteins of the DNA repair machinery. Phosphorylation of Kap1 is mediated via the phosphatidylinositol 3-kinase (PI3K)-related kinase ataxia telangiectasia mutated (ATM) (23, 24).

The mechanism by which Kap1 restricts viral replication of DNA viruses has been elucidated for a number of human herpesviruses. In the case of Kaposi’s sarcoma-associated herpesvirus (KSHV), Kap1 interacts with the KSHV latency-associated nuclear antigen (LANA) protein and has an impact on the silencing of lytic KSHV replication through recruitment of LANA/Kap1 complexes to the viral DNA (32, 33). Similarly, in Epstein-Barr virus (EBV) infection, SUMOylated Kap1 is involved in the regulation of latency by binding to the origin Lyt (oriLyt) and immediate early promoters, thereby maintaining EBV latency due to repressive functions (28, 30). For cytomegalovirus (CMV), Kap1, in complex with HP1 and the methyltransferase SETDB1, represses CMV reactivation in CD34^+^ hematopoietic stem cells (HSCs) (31). Kap1 also functions as a restriction factor in adenovirus replication and represses retroviruses and retroviral elements (25–27, 29, 34), with SUMOylated Kap1 associating with viral regulatory elements and thereby exerting repressive functions.

Here, we show that Kap1 is in a complex with MCPyV T-Ags sT and LT. Using Kap1 knockout cells, we showed that MCPyV DNA replication levels are increased in the absence of Kap1 and that partial complementation can be performed by reintroducing Kap1. In contrast to other DNA viruses, Kap1 is not recruited to MCPyV DNA and viral gene expression remains unaffected. Instead, our data obtained by analyses of normal human dermal fibroblast (nHDF) cells transfected with religated MCPyV genome or infected with MCPyV suggest that MCPyV DNA replication induces genotoxic stress followed by ATM-mediated phosphorylation of Kap1, a phenotype that is not induced by LT overexpression alone. Consequently, MCPyV-replicating nHDF cells arrest in G_2_ and undergo senescence. We hypothesize that Kap1-induced senescence is a host restriction mechanism against MCPyV replication in nHDF cells.

## RESULTS

### Kap1 interacts with the early gene products of MCPyV.

**FIG 1:**
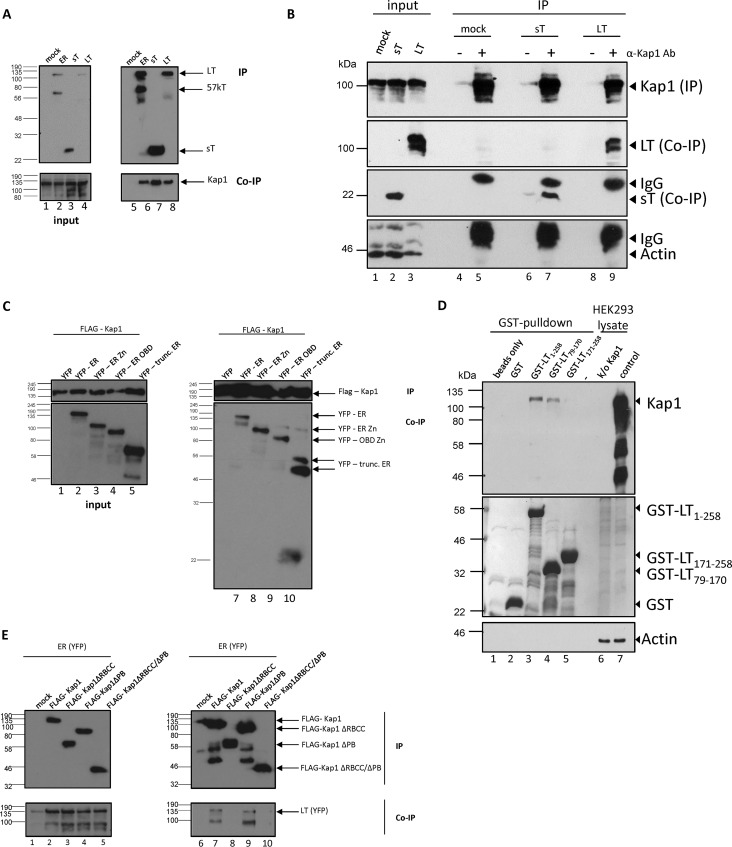
Endogenous Kap1 is in a complex with MCPyV T antigens. (A) Endogenous Kap1 coprecipitated with MCPyV T antigens (sT and LT) in H1299 cells. Cells were transiently transfected with FLAG-tagged early region (ER), FLAG-tagged LT-Ag (LT), or FLAG-tagged sT-Ag (sT). At 48 h p.t., cell lysate experiments were performed, FLAG-tagged T proteins precipitated with M2 beads (upper blot, IP), and coprecipitated endogenous Kap1 was detected with an anti-Kap1 antibody (lower blot, Co-IP). (B) Endogenous Kap1 coprecipitated with untagged MCPyV T antigens. Cells were transiently transfected with untagged LT or sT; using methods similar to those used in the experiments whose results are shown in panel A, cell lysates were prepared, endogenous Kap1 was precipitated with an anti-Kap1 antibody, and coprecipitating T-Ags were detected with Cm2B4 Ab (LT) or 2T2 Ab (sT). (C) The N-terminal region of MCPyV LT-Ag interacts with Kap1. H1299 cells were transiently transfected with FLAG-tagged Kap1 in the presence of YFP-tagged MCPyV early region constructs. Kap1 was precipitated with an anti-FLAG antibody (IP), and coprecipitated T antigens were detected using a GFP antibody (Co-IP). trunc., truncated. (D) N-terminal region aa 1 to 258 is essential for Kap1 binding. GST pulldown experiments were performed with GST-tagged LT deletion constructs (20). Proteins extracts from HEK293 cells were incubated with GST-tagged proteins, and bound Kap1 protein was visualized by Western blotting using Kap1-specific antibody (upper panel). The middle panel illustrates the amount of GST fusion proteins used in the experiment as illustrated by Coomassie staining. GST pulldown assay data show that the DNAJ domain contributed to LT binding; however, aa 171 to 258 also mediated Kap1 binding. (E) The RBCC domain of Kap1 interacts with MCPyV LT-Ag. H1299 cells were transiently transfected with FLAG-tagged Kap1 expression constructs in the presence of the YFP-tagged MCPyV early region. Ectopically expressed Kap1 proteins were precipitated with an anti-FLAG antibody (IP), and precipitating T proteins were detected by the use of Cm2B4 antibody (Co-IP).

We recently identified chromatin-associated protein Kap1 as a putative cellular binding partner of the MCPyV early gene products by mass spectrometry (22). To confirm this initial observation and to characterize which early gene product interacts with Kap1, we performed coimmunoprecipitation (Co-IP) experiments in H1299 cells ectopically expressing the FLAG-tagged early region (ER) protein, FLAG-tagged LT protein, or FLAG-tagged sT protein (Fig. 1A). Furthermore, we show that this coprecipitation was independent of protein tags (Fig. 1B). To specify the protein regions required for Kap1 LT interaction, we performed Co-IPs in H1299 cells overexpressing FLAG-tagged Kap1 protein and yellow fluorescent protein (YFP)-tagged MCPyV full-length early gene region (ER) protein or ER protein with the truncating mutations that follow within the LT-Ag open reading frame (ORF) and lead to early termination after the Zinc finger domain, after the origin binding domain (OBD), or upstream of the OBD (as can be found in MCC cells). Co-IPs performed with an anti-FLAG antibody (Ab) showed that the full-length as well as all the C-terminally truncated MCPyV ER products interacted with the FLAG-tagged Kap1 protein, suggesting that residues within the N-terminal regions that are shared between LT and sT are important for Kap1 binding of T-Ags (Fig. 1C). To clarify whether the DNAJ domain (amino acids [aa] 1 to 70) in the N-terminal regions of both LT and sT (11) confers Kap1 binding, we performed glutathione *S*-transferase (GST) pulldown experiments with HEK293 cell lysates and bacterially expressed LT deletion mutants N-terminally fused to GST encompassing aa 1 to 258, aa 171 to 258, or aa 79 to 170 (20). While we observed a Kap1 binding in the case of the deletion mutant encompassing aa 1 to 258 and, to a lesser extent, in that encompassing aa 79 to 170, we did not observe binding to endogenous Kap1 in that encompassing aa 171 to 258 (Fig. 1D). Reciprocally, Co-IPs addressing which Kap1 domain is important for LT binding revealed that the Kap1 N-terminal RBCC domain is essential for LT precipitation (Fig. 1E).

### Kap1 depletion increases MCPyV DNA replication.

**FIG 2:**
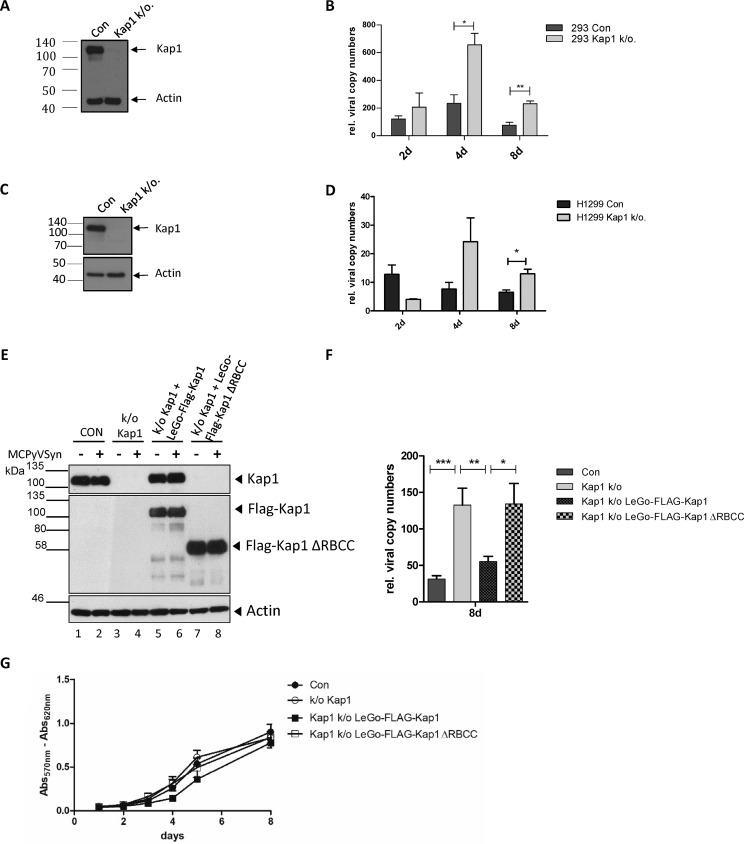
Kap1 restricts MCPyV replication. (A to D) Kap1 was deleted by the use of CRISPR/Cas9 technology in HEK293 cells (A and B) and H1299 cells (C and D). (A and C) Western blots of control cells (Con) and Kap1 knockout (k/o.) cells. (B and D) qPCR results of MCPyV DNA replication assays. qPCR results are illustrated as MCPyV copy numbers measured by the use of VP1 normalized to GAPDH. Shown are means and standard deviations (SD) of results from three individual experiments. Unpaired *t* tests were performed for statistical analysis. rel., relative; 2d, day 2; 4d, day 4; 8d, day 8. (E to G) HEK293 Kap1 knockout cells were rescued for Kap1 expression by transduction of LeGo-FLAG-Kap1 and a mutant devoid of RBCC domain, LeGo-FLAG-Kap1ΔRBCC. (E) Immunoblots of cell lysates from control cells, Kap1 knockout cells, and cells transduced with LeGo-FLAG-Kap1 (lanes 5 and 6) or LeGo-FLAG-Kap1ΔRBCC (lanes 7 and 8) from day 8 of a MCPyV replication assay. (F) MCPyV DNA replication assays in the cells represented in panel E; shown are MCPyV copy numbers normalized to GAPDH from eight independent experiments. (G) MTT assays of cells used in the MCPyV replication assays performed as described for panels E and F. Data represent eight independent experiments. Two-way statistical analysis of variance (ANOVA) revealed no significant differences between cell lines. *, *P* < 0.05; **, *P* < 0.01; ***, *P* < 0.001.

To evaluate the consequences of Kap1/T protein interaction, we performed MCPyV DNA replication assays using a semipermissive system (12, 15, 16, 35, 36) in cells positive or negative for Kap1 expression. We generated HEK293 cells (Fig. 2A and B) or H1299 cells (Fig. 2C and D) in which Kap1 was deleted by CRISPR/Cas9 (Fig. 2A and C). The levels of proliferation of Kap1 knockout cells did not significantly differ from the levels seen with the control (CON) cells (Fig. 2G). We then transfected Kap1 knockout and parental control cells with recircularized MCPyV genomes, isolated the genomic DNA at the indicated time points, and performed DpnI digestion and quantitative PCR (qPCR) analysis of replicated DNA. Intriguingly, we observed a significant increase in MCPyV DNA replication in Kap1 knockout cells at 4 and 8 days posttranslation (p.t.) (Fig. 2B and D). This phenotype of increased MCPyV DNA replication could be partially reversed by lentivirus-mediated reexpression of full-length Kap1 in Kap1 knockout cells but not by reexpression of a Kap1 protein missing the RBCC domain, which is responsible for LT binding (Fig. 2E to G).

### Subcellular Kap1 localization does not change in cells replicating MCPyV.

**FIG 3:**
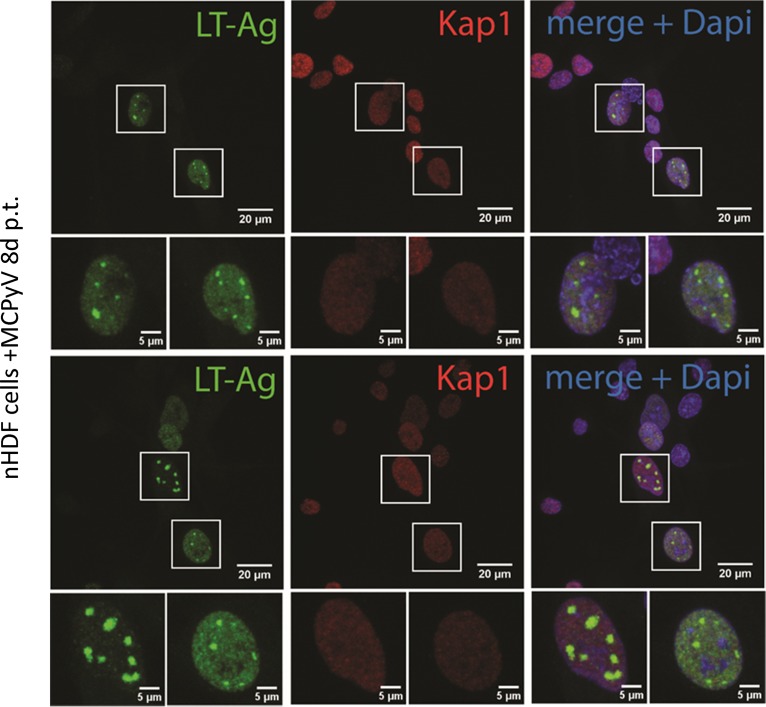
Kap1 cellular localization does not change in cells actively replicating MCPyV. nHDF cells were transfected with religated MCPyV genome by electroporation. At day 8 p.t., LT (green) and Kap1 (red) expression was followed by immunohistochemical staining.

To address whether LT and Kap1 colocalize in cells actively replicating MCPyV, we performed immunofluorescence (IF) staining in nHDF cells transfected with MCPyV genome. While nHDF cells supported MCPyV DNA replication to a higher extent than PFSK-1 cells (see Fig. S1A to C in the supplemental material), we did not observe significant differences in Kap1 localization between cells positive or negative for LT expression (Fig. 3). In both groups, the calculation of the Pearson correlation coefficient indicated low to moderate colocalization, with means of 0.56675 in cells with diffuse LT staining and 0.56675 in cells clearly showing an LT replication center. Kap1 localization was characterized by diffuse and slightly granular nuclear staining results.

10.1128/mBio.00142-20.1 FIG S1 Primary nHDF cells show significantly higher levels of MCPyV replication than immortalized, established cell lines. (A and B) nHDF cells (A) or PFSK-1 cells (B) were transfected with religated MCVSyn (15) with similar levels of transfection efficiency in the two cell lines. Immunofluorescence staining against LT protein was performed at 2 days p.t. (C) MCPyV DNA replication after 2, 4, and 8 days p.t. determined by qPCR. MCPyV copy numbers are normalized to GAPDH DNA (shown are the mean values and SD of results from three independent experiments). (D) relative MCPyV copy numbers in nHDF cells transfected with MCPyV genome 2 and 7 d p.t. Cells were harvested at the indicated time points, and DNA was isolated from cells and filtered supernatant of cells. (E, F) MCPyV gene expression does not change in the presence/absence of Kap1. HEK293 cells (E), H1299 cells (F) control (Con) and equivalent Kap1 knockout cells were transfected with the religated MCPyV genome. After the indicated time points MCPyV early (LT) and late (VP1) transcripts were determined by RT-qPCR and normalized to GAPDH transcripts and MCPyV genome copy numbers. Shown are the means and SD of results from three independent experiments (unpaired *t* test) for the early transcripts (late transcripts were below detection limit). Download FIG S1, TIF file, 2.1 MB. Copyright © 2020 Siebels et al. 2020 Siebels et al. This content is distributed under the terms of the Creative Commons Attribution 4.0 International license.

### Kap1 is not recruited to viral DNA and has no direct influence of LT binding to the viral ori.

**FIG 4:**
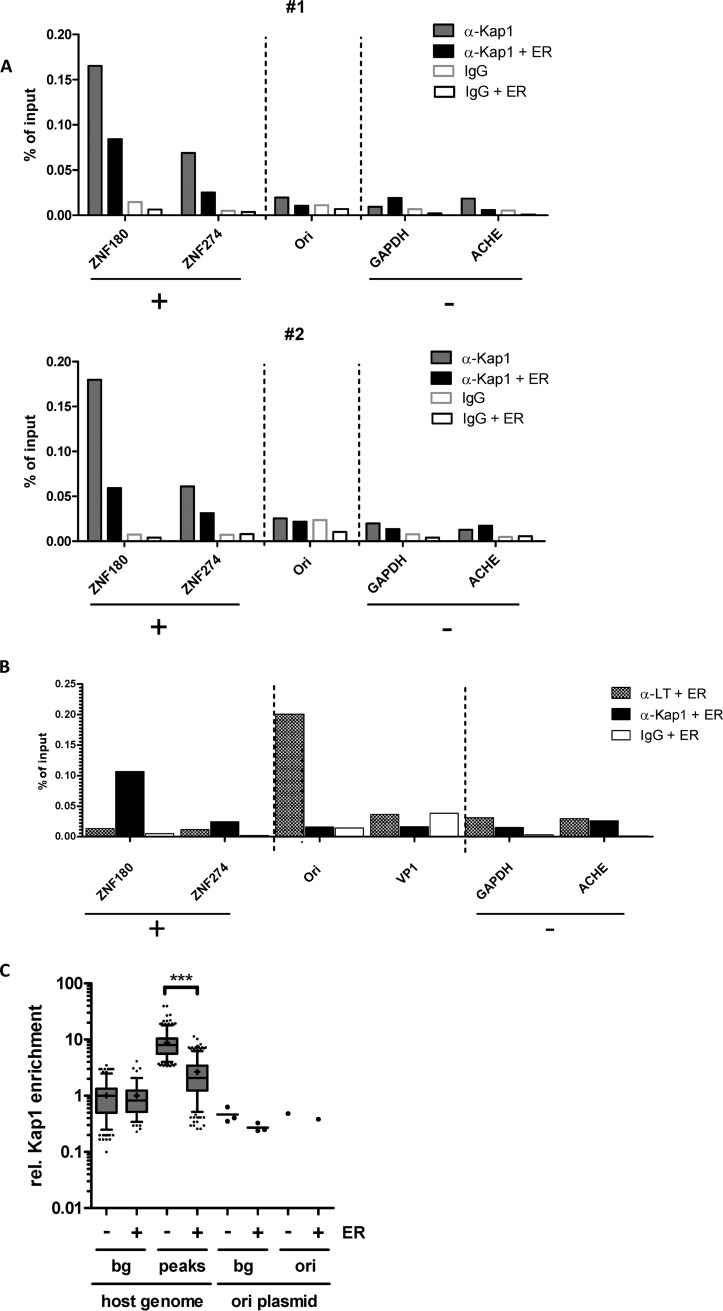
Kap1 does not bind to the MCPyV genome. HEK293 cells transiently expressing LT-Ag or control plasmid were additionally transfected with a plasmid containing the MCPyV origin of replication (ori). (A and B) ChIP experiments were performed using the anti-Kap1 antibody (Abcam ab10483) (A) or anti-LT antibody (Santa Cruz sc136172) (B). (A) Representation of two independent Kap1 ChIP experiments (experiments 1 and 2); shown are ChIP-qPCR results representing positive-control (ZNF180 and ZNF274) and negative-control (ACHE and GAPDH) regions for Kap1 binding. (B) ChIP PCR experiment performed similarly to the experiments whose results are shown in panel A, with an additional ChIP performed using the anti-LT Ab Cm2B4. A positive-control region of LT binding is represented by the origin of replication (Ori), while VP1 represents a negative-control region. (C) ChIP-seq analysis (*n* = 1) of Kap1 binding to cellular and ori-containing plasmid DNA in either control cells (-) or ER-expressing HEK293 cells (+). Kap1 peaks (*n* = 456) were detected on the host genome using MACS2. The cellular ChIP background (bg) level was determined using the same amount of matched control sites generated by EaSeq. All data representing host and ori results are represented as relative levels of read count enrichment in the respective region windows over the input sample. Host background levels in both samples were set to a value of 1 to enable comparability of data from ER-expressing and control cells as well as host loci and plasmids. The statistical significance of data representing differential Kap1 binding to host cell loci was determined using a two-tailed *t* test. Three background regions on the ori plasmid (scatterplot; data represent means and standard errors of the means [SEM]) and the ori region itself were used to analyze the relative levels of Kap1 binding to the ori.

Since previous studies had demonstrated Kap1 recruitment to herpesviral control regions (CRs) such as the KSHV replication and transcription activator (RTA) promoter (32), we examined the binding of Kap1 at the MCPyV origin of replication (ori). We performed chromatin immunoprecipitation-qPCR (ChIP-qPCR) and ChIP sequencing (ChIP-seq) with an anti-Kap1 antibody and a control antibody in HEK293 cells transiently transfected with an MCPyV ER expression construct and an additional plasmid carrying the MCPyV ori region (Fig. 4). The precipitated DNA was quantified by qPCR with primers specific for the ori region, cellular promoter regions known to be bound by Kap1 in HEK293 cells (positive controls; ZNF180 and ZNF274), or regions in the cellular GAPDH (glyceraldehyde-3-phosphate dehydrogenase) and ACHE genes (negative controls) (37). As shown in Fig. 4, Kap1 levels were significantly enriched at the ZNF180 and ZNF274 promoter regions compared to the negative controls. In contrast, we observed no enrichment at the viral ori, regardless of whether LT was present or absent (Fig. 4). Interestingly, we observed decreased Kap1 binding at the cellular control regions in cells overexpressing the ER protein (Fig. 4A and C). We included LT ChIP PCR as a control to enrich for DNA regions located on relatively small, circular DNAs (Fig. 4B). We confirmed the enrichment of Kap1 at cellular promoter regions by ChIP-seq and observed statistically significant enrichment of Kap1 at host promoter regions compared to the viral ori (Fig. 4C). These results demonstrate that Kap1 is not recruited to the viral noncoding control region (NCCR). In line with this finding, we did not observe an effect of Kap1 on the expression of early or late viral transcripts as indicated by qPCR performed in viral replication assays in cells positive or negative for Kap1 expression (Fig. S1E and F).

To investigate whether the restriction in MCPyV DNA replication was due to modulation of LT binding to the viral ori region, we performed ChIP-qPCR experiments with an anti-LT Ab (Cm2B4) in HEK293 cells and HEK293 Kap1 knockout cells transiently transfected with LT and the MCPyV genome. As expected, we observed LT enrichment at the viral ori (Fig. S2A); however, this enrichment remained unchanged in the presence or absence of Kap1 (Fig. S2A and B). We obtained similar results by performing electrophoretic mobility shift assays (EMSAs) in HEK293 cells and HEK293 Kap1 knockout cells (Fig. S2C) and DNA-protein interaction–enzyme-linked immunosorbent assays (DPI-ELISAs) using streptavidin-binding-peptide (SBP) tag-purified LT from HEK293 cells and HEK293 Kap1 knockout cells (Fig. S2D to F).

10.1128/mBio.00142-20.2 FIG S2 Kap1 does not affect the binding of LT to the viral origin of replication. (A, B) Chromatin-IP of LT-Ag (Cm2B4) in HEK293 Con or Kap1 knockout cells transiently transfected with an LT-Ag expression construct and religated MCPyV genome. Shown are the means and standard deviations (SD) of results from three experiments (unpaired *t* test). (C) Electrophoretic mobility shift assay (EMSA) using nuclear extract from HEK293 Con and HEK293 Kap1 knockout cells in the presence of a ^32^P-radiolabeled 80-bp ori probe. LT-specific band shifts are labeled with a number sign (#). (D and E) DNA-protein interaction–ELISA using LT protein purified from HEK293 Con cells (D) or Kap1 knockout cells (E), with results shown by Coomassie staining. (F) LT-Ag binding to the ori relative to an ori scrambled probe (with similar levels of GC content but with shuffled GRGCC motifs). Shown are the means and SD of results from three independent experiments (unpaired *t* test). Download FIG S2, TIF file, 1.2 MB. Copyright © 2020 Siebels et al. 2020 Siebels et al. This content is distributed under the terms of the Creative Commons Attribution 4.0 International license.

### MCPyV DNA replication induces Kap1 phosphorylation in cells transfected with MCPyV genome and cells infected with MCPyV.

**FIG 5:**
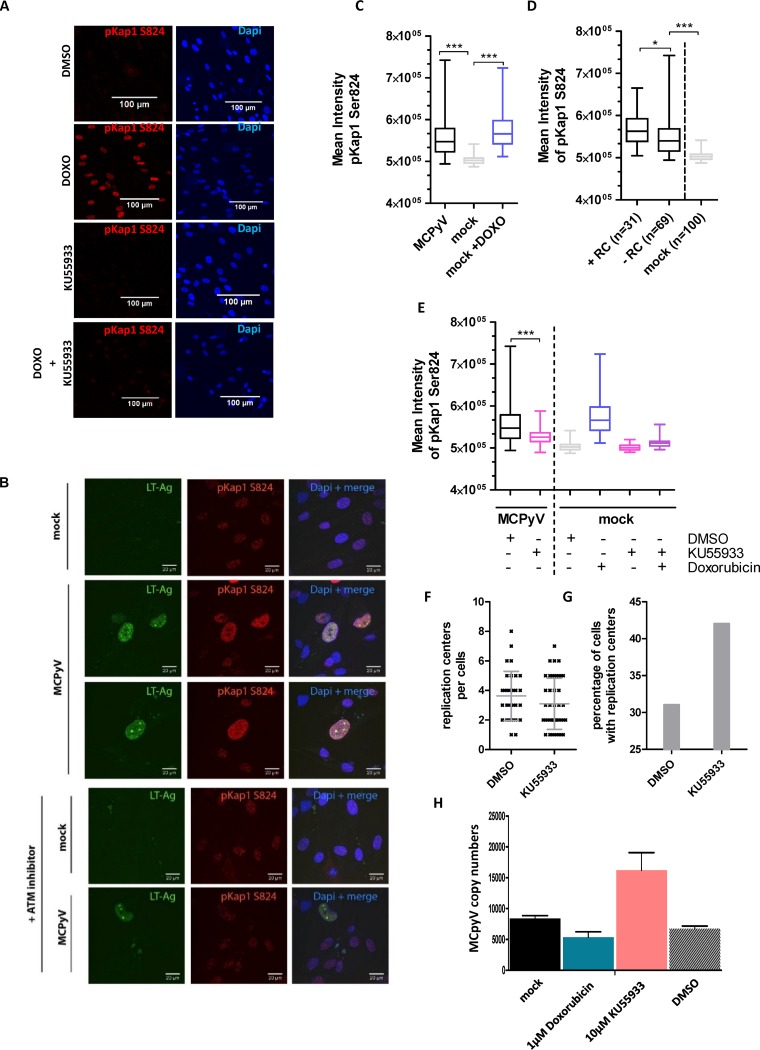
MCPyV replication induces Kap1 phosphorylation on serine 824. (A) Immunofluorescence staining against pKap1 S824 on nHDF cells treated with dimethyl sulfoxide (DMSO), doxorubicin (DOXO), ATM inhibitor KU55933, and doxorubicin plus KU55933. (B) nHDF cells were transfected with religated MCPyV genome by electroporation. At 8 days p.t., levels of LT expression and the phosphorylation status of Kap1 were determined by immunofluorescence using confocal microscopy. (C) Mean phospho-Kap1 intensity levels were quantified using ImageJ. As a positive control, Kap1 phosphorylation was induced by the use of 1 μM doxorubicin for 24 h. The Mann-Whitney test was applied to calculate the statistical significance of the mean levels of phospho-Kap1 intensity for 100 cells. (D) Phosphorylation of Kap1 S824 was increased in cells with MCPyV replication centers (RC). The Mann-Whitney test was applied to calculate the statistical significance of the differences in mean pKap1 S824 intensity for *n* = 31, *n* = 69, and *n* = 100 cells. (E) nHDF cells were transfected with the religated MCPyV genome by electroporation. At 7 days p.t., cells were treated with 1 μM doxorubicin and 10 μM ATM inhibitor KU55933 for 24 h. At 8 days p.t., the phosphorylation status of Kap1 was analyzed by immunofluorescence. Mean pKap1 S824 intensity levels were quantified using ImageJ. The Mann-Whitney test was applied to determine statistical significance (100 cells). (F to H) nHDF cells transfected with religated MCPyV genome and treated with doxorubicin or KU55933 as described for panel E. (F and G) Cells were analyzed by immunofluorescence, and numbers of replication centers per cell (F) and percentages of cells positive for LT replication centers (G) were quantified. (H) DpnI-sensitive viral DNA replication assays of the cells described in the panel F and G legends.

Kap1 abundance and functions are regulated by posttranslational modifications. Kap1 SUMOylation and subsequent CHD3 and SETDB1 recruitment result in a repressive function of Kap1. Kap1 can also be phosphorylated, mainly at serine 824 and serine 473 in the context of DNA damage responses (Fig. 5A). Phosphorylation at these residues counteracts Kap1 SUMOylation, resulting in remodeling of heterochromatic regions and increased accessibility to proteins of the DNA damage response pathway.

**FIG 6:**
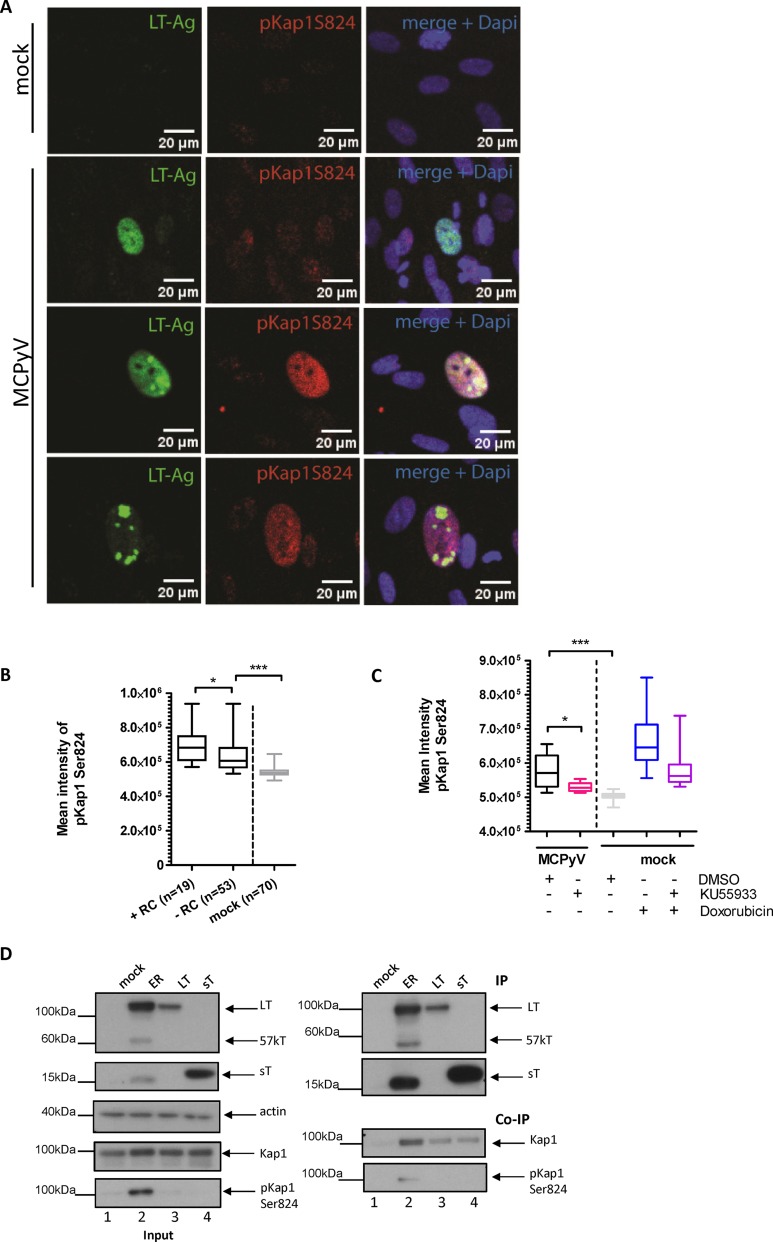
(A to C) Induction of Kap1 S824 phosphorylation in cells infected with MCPyV. nHDF cells were infected with MCPyV (2 × 10^9^) (14). (A) Immunofluorescence images of anti-LT (Cm2B4) and anti-Kap1 S824 staining analyzed by confocal microscopy 5 weeks p.i. (B) Data represent results of Mann-Whitney tests performed to calculate the statistical significance of differences in mean pKap1 intensity between cells positive for LT expression with replication centers, cells positive for LT expression but no visible LT replication center, and control cells at 5 weeks p.i. (C) At 14 days p.i., nHDF cells were treated with 1 μM doxorubicin and/or 10 μM ATM inhibitor KU55933 for 24 h. Mean phospho-Kap1 S824 intensity levels were quantified using ImageJ. The Mann-Whitney test was used to calculate the statistical significance of results determined for *n* = 10 (column 1), 14 (column 2), 30 (column 3), 30 (column 4), and 30 (column 5) cells. (D) pKap1S824 coprecipitated with MCPyV LT antigen. HEK293 cells were transfected with FLAG-tagged MCPyV early viral region-expressing LT and sT (lanes 2) and with expression constructs expressing FLAG-tagged LT (lanes 3) or sT (lanes 4). At 48 h p.t., cell lysates with FLAG-tagged T antigens were precipitated using M2 beads (right, upper panels) and coprecipitated endogenous Kap1 or phosho-Kap1 protein (right, lower panels) was detected by immunoblotting.

To analyze whether the phosphorylation levels of Kap1 change in cells actively replicating MCPyV, we transfected primary nHDF cells with religated MCPyV genomes. At 8 days p.t., we determined pKap1 S824 levels by immunofluorescence. We detected that cells positive for LT showed a significant increase in the levels of pKap1 S824 (Fig. 5B and C). Additionally, upon closer inspection of the LT-positive fraction, we found the levels of pKap1 S824 to be significantly higher in cells with distinct and large MCPyV replication centers than in those with a more uniform staining pattern, suggesting that ongoing DNA replication rather than LT expression alone induces Kap1 phosphorylation (Fig. 5D). This notion is furthermore supported by the fact that Kap1 phosphorylation was also observed in nHDF cells infected with a replication-competent MCPyV (Fig. 6A and B) but not in nHDF cultures transduced with LT or sT expression constructs alone (Fig. S3C and D).

10.1128/mBio.00142-20.3 FIG S3 Ectopic MCPyV T-Ag expression does not induce phosphorylation of Kap1 on S824 in nHDF cells. (A and B) Immunofluorescence assay using anti-Kap1 antibody (green) and anti-phoshoKap1 S824 antibody (red) in H1299 control cells (A) and H1299 cells depleted for Kap1 (B). The lower panels show the cells treated with doxorubicin to induce Kap1 phosphorylation. (C) nHDF cells were transduced with LeGo-iG (Mock), LeGo-iG-LT (LT), LeGo-iC2-sT (sT), or LeGo-iG-LT and LeGo-iC2-sT (LT+sT). At 4 days p.t., cell lysates were analyzed by Western blotting for the expression of Kap1, pKap1S824, LT and sT. (D) nHDF cells were electroporated with LeGo-iG-LT and analyzed by confocal microscopy (2 days p.t.) for the expression of LT and pKap1 S824. (E) Western blotting of the nHDF cells shown in panel D together with HEK293 cells for comparison of pKap1S824 induction results. Download FIG S3, TIF file, 1.8 MB. Copyright © 2020 Siebels et al. 2020 Siebels et al. This content is distributed under the terms of the Creative Commons Attribution 4.0 International license.

10.1128/mBio.00142-20.4 FIG S4 Decreased sumoylation of endogenous Kap1 in the presence of T antigen expression. Two independent experiments (panels A and B) were performed with HeLa cells (Par); HeLa sumo1 cells (included only in panel A) and HeLa sumo2 cells stably expressing His-tagged sumo1 or sumo2 (39) were transfected with a control vector or a plasmid containing the MCPyV early region (ER). At 2 days p.t., cells were lysed and His-Sumo-1 and His-Sumo2 were precipitated using Ni-NTA agarose beads. Precipitated protein was analyzed by Western blotting. Immunoblotting with an anti-Kap1 antibody shows the amount of endogenous Kap1 protein in the input and the amount of sumo-conjugated Kap1 protein in the precipitated fractions (Ni-NTA IP). Download FIG S4, TIF file, 1.6 MB. Copyright © 2020 Siebels et al. 2020 Siebels et al. This content is distributed under the terms of the Creative Commons Attribution 4.0 International license.

10.1128/mBio.00142-20.5 FIG S5 MCPyV mutant K331A does not bind to the viral ori DNA. (A) Western blotting of HEK293 cells ectopically expressing empty control plasmid (mock), wild-type LT-Ag (wt LT), or LT-Ag containing a point mutation at position 331 resulting in an amino acid exchange of lysine to alanine (LT K331A). (B) DPI-ELISA using nuclear extracts from HEK293 cells transiently expressing LT-Ag as shown in panel A and biotinylated DNA probes corresponding to the minimal ori region, an ori scramble probe, and a negative-control DNA probe corresponding to VP1 (MCVSyn; nt 2553 to 2632). Shown are the means and SD of results from three independent experiments (unpaired *t* test). (C) MCPyV *in vitro* replication assays in HEK293 cells. HEK293 cells were transfected with religated MCPyV genome (wild-type [wt]) and with religated MCPyV genome with aa 331 K-to-A mutation. After 1, 4, and 8 days p.t., cells were harvested and DNA was extracted, and after DpnI digestion, MCPyV genome copy numbers were determined by qPCR. MCPyV copy numbers are normalized to GAPDH. Shown are the copy numbers relative to those determined at day 1 of three independent experiments. Download FIG S5, TIF file, 0.8 MB. Copyright © 2020 Siebels et al. 2020 Siebels et al. This content is distributed under the terms of the Creative Commons Attribution 4.0 International license.

Since Kap1 phosphorylation can be induced by ATM and ATM is substantially activated in cells replicating MCPyV (38), we analyzed Kap1 phosphorylation in the presence or absence of an ATM inhibitor, KU55933. Results of experiments in nHDF cells transfected with replication-competent MCPyV genomes (Fig. 5D and E) or infected with MCPyV particles (Fig. 6C) indeed indicate that phosphorylation of Kap1 on serine 824 is dependent on ATM. Furthermore, when we treated cells with an ATM inhibitor, we observed an increase in the abundance of cells with MCPyV DNA replication centers together with an increase of MCPyV copy numbers, indicating that ATM-dependent phosphorylation of Kap1 is critical for MCPyV replication (Fig. 5F to H).

On the basis of our observations that the viral early proteins coprecipitated Kap1 and that cells transduced with replication-competent MCPyV genomes induced pKap1, we subsequently analyzed whether LT and sT also coprecipitate with phosphorylated Kap1. Due to insufficient numbers of cells being positive for MCPyV in *in vitro* replication and MCPyV infection, we performed these experiments in HEK293 cells overexpressing the early region (ER), LT, or sT protein (Fig. 6D). Interestingly, the HEK293 cells, in contrast to the nHDF cells, showed pKap1S824 induction in response to viral ER expression (Fig. 6D, lane 2; see also Fig. S6E). Immunoprecipitation of FLAG-tagged early viral proteins showed that pKap1S824 was coprecipitated only in the case of viral ER expression, which also results in significantly higher LT expression levels and pKap1S824 induction.

10.1128/mBio.00142-20.6 FIG S6 Transcriptome analysis of HEK293, HEK293 ER, HEK293 Kap1 knockout, and HEK293 Kap1 knockout ER cells. (A) Principal-component analysis (PCA) of HEK293 cells (wt mock, blue), HEK293 Kap1 knockout cells (Kap1 knockout mock, light blue), HEK293 Kap1 knockout ER cells (Kap1 knockout ER, pink), and HEK293 ER cells (wt ER, green); (B) Normalization by DeSeq2. The color coding corresponds to the color code used in PCA. (C) Total number of reads per individual replicate. (D) KEGG pathway analysis. All significantly upregulated genes (log_2_ fold change greater than or equal to 1; FDR greater than 0.1) and all significantly downregulated genes (log_2_ fold change less than or equal to −1); FDR less than 0.1) from three independent experiments were analyzed using DAVID (54, 55). Columns show the results for genes regulated by Kap1, genes regulated by MCPyV T-Ag, and genes that were regulated by both Kap1 and MCPyV T-Ags. Columns 1 to 6 correspond to the comparisons illustrated in Fig. 8A. Shown are the top five KEGG pathways. Download FIG S6, TIF file, 1.6 MB. Copyright © 2020 Siebels et al. 2020 Siebels et al. This content is distributed under the terms of the Creative Commons Attribution 4.0 International license.

Since Kap1 function is regulated by phosphorylation and SUMOylation, which counteract each other, we next analyzed potential changes of Kap1 SUMOylation levels. For this purpose, we used HeLa cells stably expressing His-SUMO1 or His-SUMO2 (39). Western blotting of nickel-nitrilotriacetic acid (Ni-NTA)-purified His-SUMO1/2 conjugates demonstrated clear conjugation of the endogenous Kap1 protein to SUMO2, while no Kap1 complexes were retrieved from His-SUMO1 cells (Fig. S4). Transfection of HeLa His-SUMO2 cells or the corresponding parental cells with MCPyV ER or vector control (mock) resulted in decreased SUMO2-Kap1 levels (Fig. S4, lower panel, lanes 4 and 8), suggesting that expression of LT or sT or both is sufficient to reduce Kap1 sumoylation.

### MCPyV replication induces Kap1-dependent senescence in nHDF cells.

**FIG 7:**
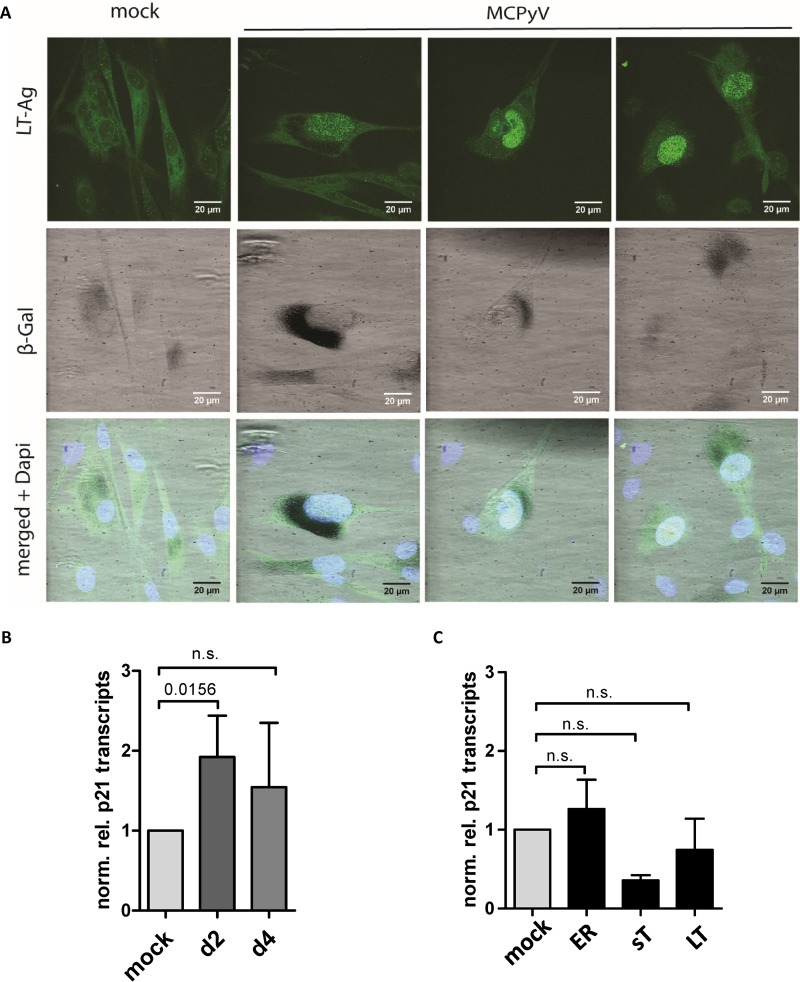
MCPyV replication induces senescence in nHDF cells. (A) β-Galactosidase (β-Gal) staining of nHDF cells transfected with religated MCPyV genome. At 10 days p.t., cells were stained for β-galactosidase and LT-Ag expression (immunofluorescence staining). (B) p21 transcription levels in nHDF cells transfected with religated MCPyV genome. p21 transcripts were quantified at the indicated time points and normalized to housekeeping genes YWHAZ and HPRT1. Shown are means and SD of results from seven replicates. For analyses of statistical significance, the nonparametric one-sample *t* test (Wilcoxon signed-rank test) was performed. n.s., not statistically significant. (C) p21 transcripts in nHDF cells transfected with expression constructs coding for LT, for sT, or for LT and sT. p21 transcripts were determined at 48 h p.t. as described for panel B.

Kap1 has recently been shown to confer senescence in cells responding to DNA damage (40). To investigate whether MCPyV replication, subsequent ATM activation, and phosphorylation of Kap1 S824 would result in a senescent phenotype, we transfected nHDF cells with replication-competent MCPyV genomes and stained for expression of β-galactosidase, a protein that functions as a marker of senescent cells, at day 10 p.t. (Fig. 7). Indeed, cells positive for LT expression showed highly increased β-galactosidase staining compared to LT-negative cells (Fig. 7A). We did not succeed in quantification of sufficient numbers of these cells for statistical analysis, since only a minor fraction of the cells supported MCPyV replication as reflected by results of analyses of replication compartments. However, as the senescent phenotype is generally accompanied by the activation of p21, we chose to investigate p21 transcript levels instead. As shown in Fig. 7, we found p21 transcript levels to be significantly increased in nHDF cells replicating MCPyV (Fig. 7B). Cells ectopically expressing LT, sT, or ER (Fig. 7C) did not exhibit p21 upregulation, again suggesting that active DNA replication induces the phenotype and not early gene expression alone.

**FIG 8:**
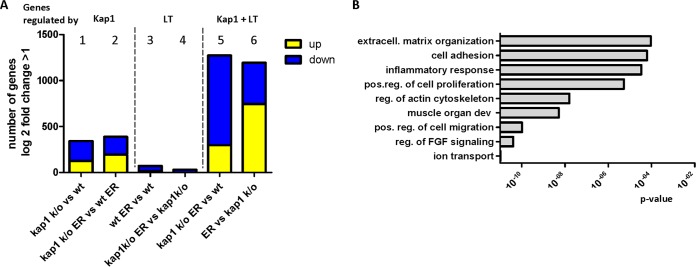
Transcripts regulated by LT-Ag in the presence and absence of Kap1. (A) Transcriptome analysis of HEK293 CON cells; HEK293 Kap1 knockout cells in the presence and absence of LT-Ag. Shown are numbers of genes which were significantly upregulated or downregulated (log_2_ fold change, >1; FDR, <0.1. (B) GO terms of genes regulated by Kap1 and MCPyV T antigens. Shown are the GO-Terms (DAVID Functional Annotation Tool [55]) with the lowest *P* value identified. cytoskelet, cytoskeleton; extracell., extracellular; FGF, fibroblast growth factor; pos.reg., positive regulation.

**FIG 9:**
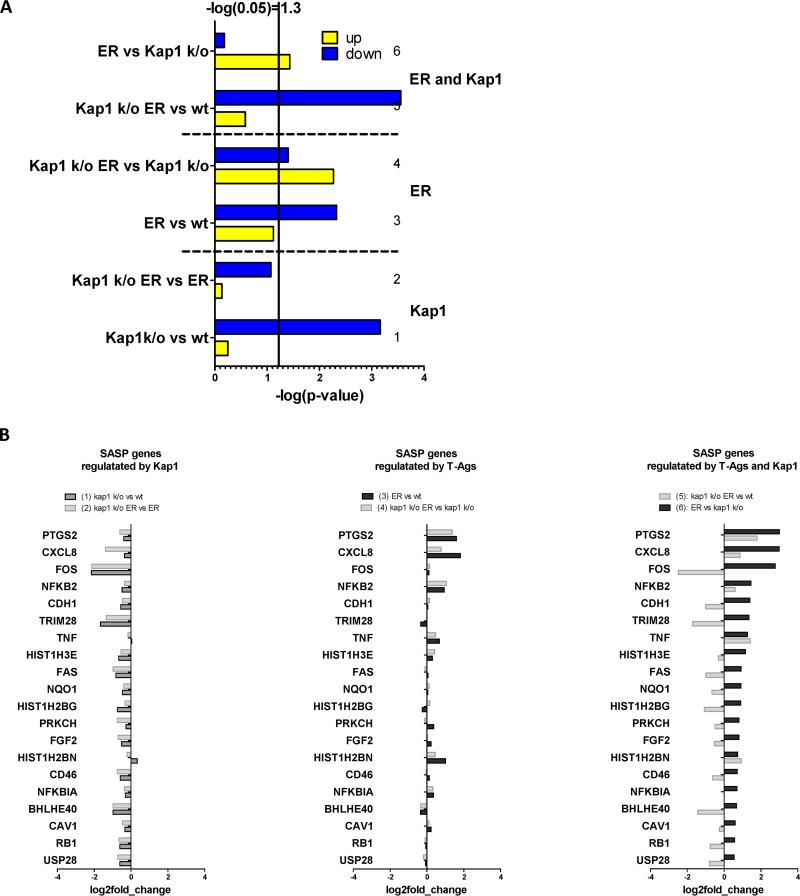
(A) Data representing 204 genes associated with senescence and a SASP were downloaded from Genecards (Table S3). To evaluate enrichment of these genes in Kap1-dependent or ER-dependent DEGs, a hypergeometric test was performed for each of comparisons 1 to 6, with 20,200 protein coding genes representing the total population and 202 senescence genes and a SASP-associated gene representing successes. The graph shows negative logarithms of the resulting *P* values. (B) The genes associated with the SASP of comparison 6 (LT versus Kap1 knockout) were ranked, and the 20 most highly differentially expressed genes are presented according to their log_2_ fold change values. Before the ranking was performed, genes with a baseMean value of <10 were rejected.

Since we did not find a direct repressive role of Kap1 with regard to viral gene expression, we aimed at elucidating the role of Kap1 by performing host transcriptome analysis. Accordingly, HEK293 cells and HEK293 Kap1 knockout cells were transiently transfected with an ER construct and at 48 h p.t., mRNA was isolated and subjected to transcriptome analyses (Fig. S6). We first focused on significant changes (log_2_ fold change of >1; false-discovery rate [FDR] of <0.1) dependent on the presence or absence of Kap1 in either ER-negative or ER-positive cells by comparing transcriptome data from HEK293 Kap1 knockout cells with data from HEK293 cells or HEK293 Kap1 knockout ER cells with HEK293 ER cells, respectively (Fig. 8A, comparisons [columns] 1 and 2). In line with previous reports (27), we found that Kap1 was involved in the repression of transcription, mainly of that of ZNF proteins (see Table S2 in the supplemental material). To look for significant changes induced by the ER proteins in either the presence or absence of Kap1, we compared HEK293 ER with HEK293 cells or HEK293 Kap1 knockout ER with HEK293 Kap1 knockout cells, respectively (comparisons 3 and 4). Gene ontology (GO) term analysis of differentially expressed genes (DEGs) in comparisons 3 and 4 (see Table S2) suggested that ER expression regulates a number of transcripts associated with the inflammatory response, the NF-κB pathway, and cell proliferation. Finally, to look for DEGs dependent on ER as well as Kap1 expression, we analyzed DEGs in HEK293 Kap1 knockout ER versus HEK293 cells or HEK293 ER versus HEK293 Kap1 knockout cells (Fig. 8B; comparisons 5 and 6; see also Fig. S6D and Table S2). GO analysis revealed that genes involved in organization of the extracellular matrix, cell adhesion, inflammatory responses, and cell proliferation are differentially expressed in a manner dependent on the presence of both Kap1 and MCPyV ER. In senescent cells, upregulation of inflammatory cytokines, chemokines, extracellular matrix remodeling factors, and growth factors contributes to the so-called senescence-associated secretory phenotype (SASP) (41–43). As shown in Fig. 9 (see also Fig. S7), we found several of the functional classes associated with SASP, in particular, cytokines and growth factors, to be upregulated in cells expressing the ER and Kap1.

10.1128/mBio.00142-20.7 FIG S7 Activation z-score of cytokines (A) and growth factors (B) predicted by Ingenuity pathway analysis (IPA). Shown are the Activation z-scores (z-score greater than or equal to 1 or less than or equal to −1). Red labeling represents activation; blue labeling displays inhibition. Download FIG S7, TIF file, 1.3 MB. Copyright © 2020 Siebels et al. 2020 Siebels et al. This content is distributed under the terms of the Creative Commons Attribution 4.0 International license.

**FIG 10:**
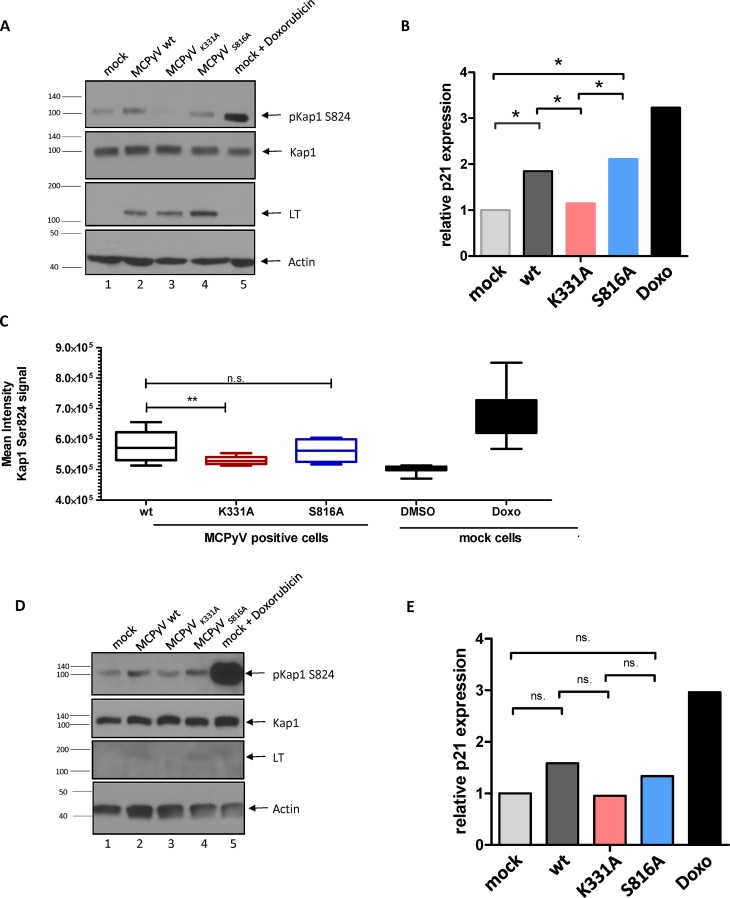
Phosphorylation of Kap1 is dependent on viral DNA replication. nHDF cells were transfected with religated MCPyV wild-type genome or mutant genomes (K331A and S816A) (38, 44). Cells were analyzed by Western blotting for the expression of LT, Kap1, and pKap1S824 at 2 days (A) and 4 days (D) p.t. (B and E) Relative p21 expression in the cells represented in panels A and D was determined as a marker for senescence by applying RT-qPCR at 2 days (B) and 4 days (E) p.t. Data were normalized to two housekeeping genes (YWHAZ and HPRT1). Shown are means and standard deviations (SD) of results from four replicates. For statistical significance, a mixed-model analysis (considering random distributions) was performed. (C) Phospo-Kap1 mean intensity signal obtained in immunofluorescence staining of the cells used as described for panels A and B.

**FIG 11:**
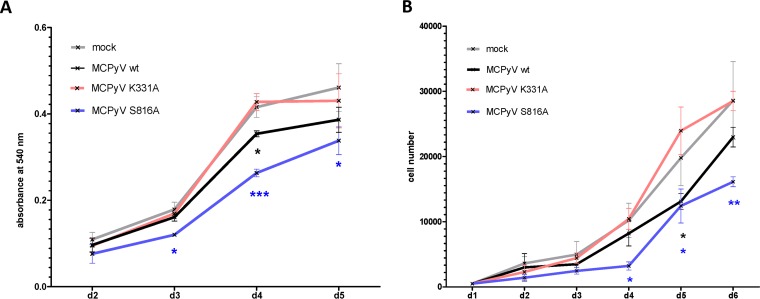
nHDF cells with replicating MCPyV genome show reduced cell proliferation. nHDF cells were transfected with religated wild-type or mutant MCPyV genomes, and proliferation was determined by the use of an MTT assay (Millipore) (A) and and automated cell counter (Bio-Rad) (B). Shown are the means and SD of results from three experiments. For statistical significance, an unpaired *t* test was performed.

**FIG 12:**
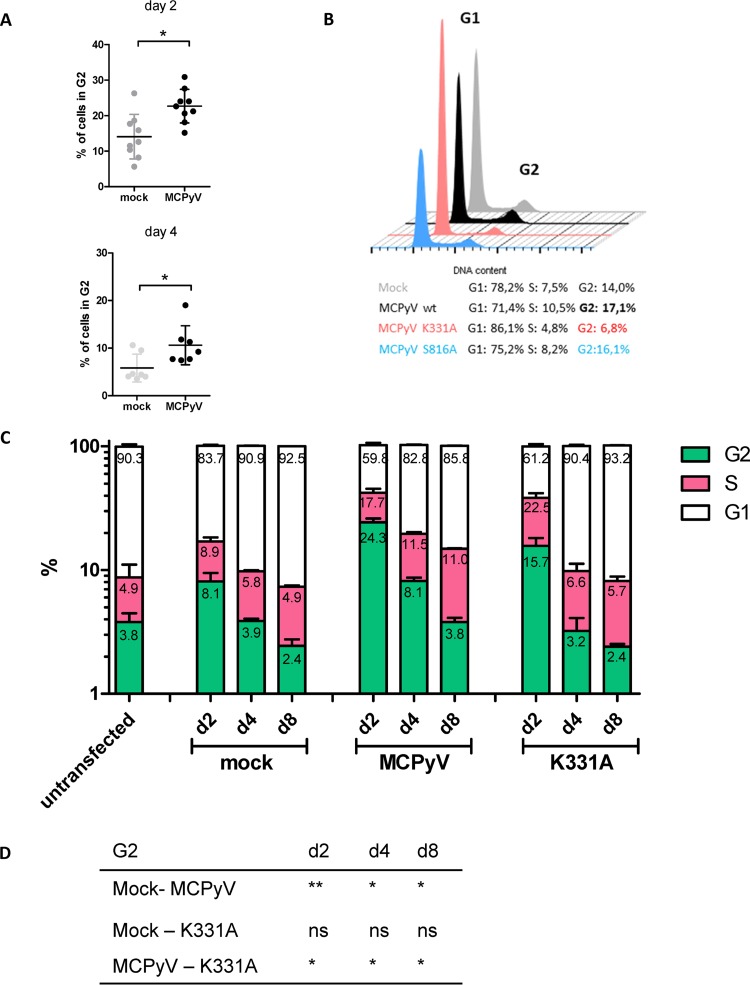
The number of cells in G_2_ arrest was increased in cells transfected with MCPyV genome. nHDF cells transfected with religated MCPyV genome were stained with propidium iodide (PI), and their levels were measured by flow cytometry. FlowJo software and the Dean-Jett-Fox algorithm was used to determine percentages of cells in G_1_, S or G_2_ phase. (A) Percentages of cells in G_2_ phase 2 and 4 days p.t. Shown are the means and SD of results from nine (day 2) and seven (day 4) experiments. For statistical significance, an unpaired *t* test was performed. (B) Percentage of cells in G_2_ phase. Cells transfected with MCPyV wt genome, MCPyV K331A genome and MCPyV S816A genome were stained with PI and analyzed by FACS at 2 and 4 days p.t. (C) nHDF cells transfected with religated MCPyV wt genome, MCPyV genome carrying the mutation K331A in the early region and MCPyV genome with the S816A mutation in the early region. Cells were treated as described for panel A, and percentages of cells are shown for the individual cell cycle stages at das 2 (d2), day 4 (d4), and day 8 (d8). (D) Statistical significance obtained by unpaired *t* test for the cells in G_2_ phase represented in panel C.

To further substantiate the hypothesis that LT-dependent viral DNA replication, but not LT expression alone, induces Kap1 phosphorylation in nHDF cells, we generated full-length viral genomes expressing an LT protein with a mutation in the DNA-binding domain (K331A) (44). Immunoblotting, DNA-protein interaction–ELISA (DPI-ELISA), and *in vitro* replication assays verified that the mutant LT-Ag was efficiently expressed but was unable to bind to the viral origin and unable to support DNA replication (Fig. S5). As shown in Fig. 10, the viral mutant was unable to induce Kap1 S824 phosphorylation (Fig. 10A, C, and D) or upregulation of p21 transcription (Fig. 10B and E). In contrast, the wild-type (wt) virus and a control virus expressing a replication-competent LT mutant (S861A) that was previously shown to exhibit lower levels of genotoxic stress in C33A cells (38) were able to efficiently induce phosphorylation of Kap1 S824 and p21 activation. Given previous observations of a growth-inhibiting phenotype mediated by expression of full-length LT in different cell types (18, 36, 38), we evaluated changes in the proliferation rates of nHDF cells transfected with wt or mutant MCPyV genomes. As shown in Fig. 11, cultures transfected with wt genomes or the S8631 mutant, but not those transfected with the K331A mutant, exhibited significantly lower proliferation rates than were seen with mock-transfected fibroblasts (Fig. 11). These data are furthermore supported by cell cycle analyses (Fig. 12). While we observed a clear accumulation of cells in G_2_ in nHDF cells transfected with replication-competent MCPyV genomes (Fig. 12A to C), this cell cycle arrest phenotype was less prominent in cells receiving the replication-incompetent MCPyV K331A genome. Interestingly, the replication-competent MCPyV S816A genome, which was previously shown to be less genotoxic than the wt genome (38), showed a significantly reduced proliferation rate and a slight increase in cell numbers in G_2_ (Fig. 12B).

## DISCUSSION

Here, we demonstrate that chromatin-associated factor Kap1/TRIM28 serves a restriction factor for MCPyV in primary human dermal fibroblasts, a cell type previously identified as a putative primary target of MCPyV infection *in vivo* (14). We identified Kap1 as a protein coprecipitating with the early gene products LT and sT. We show that cells devoid of Kap1 showed increased MCPyV DNA replication. This phenotype is independent of cell proliferation, since we did not observe a significant difference in the levels of proliferation in HEK293 or H1299 cells devoid of Kap1 compared to control cells. Increased MCPyV DNA replication in the absence of Kap1 was able to be partially reversed by overexpressing Kap1 in Kap1 knockout cells. However, a mutant that is devoid of the RBCC domain in Kap1, which is responsible for the interaction with the T-Ags, no longer rescued the observed phenotype.

Kap1 has previously been shown to repress viral transcription of several herpesviruses (KSHV, EBV, and CMV) but also that of parvoviruses, e.g., adeno-associated virus (AAV) (28–33, 45). For KSHV and CMV, Kap1 regulates latency by inducing the repressive histone modification H3K9me3 at lytic genes, while latent genes stay free of repressive marks (31, 32). In the early phase of KSHV infection, LANA (latency-associated nuclear antigen) recruits Kap1 to the KSHV genome to shut down lytic gene expression. Similarly, Kap1 restricts AAV replication by recruiting histone methyltransferases and depositing H3K9 marks at viral promoters. When Kap1 is depleted, these viruses show increased viral replication and viral gene expression.

Differently from the results described above, we identified an indirect mechanism by which Kap1 protects the cell against damage induced by MCPyV DNA replication. We have not observed Kap1 recruitment to the MCPyV ori, which contains the regulatory elements of early and late gene expression. By ChIP-qPCR, we have not observed an enrichment of Kap1 at viral regulatory regions, which is in line with the unchanged gene expression of MCPyV early or late region in cells with or without Kap1 expression. The fact that our ChIP-qPCR, EMSA, and DNA-protein interaction–ELISA results invariably showed no significant differences with respect to LT binding to viral DNA furthermore suggests that Kap1 does not simply interfere with LT recruitment to the viral ori to repress replication. Although we observed coprecipitation of LT with Kap1 and, to lesser extent, also with phosphorylated Kap1 in established cell lines and primary cells, our ChIP experiments did not show an enrichment of Kap1 at the origin of replication bound by LT. Whereas LT, when bound to the viral ori, forms a dodecamer, consisting of two hexamers in head-to-head orientation, LT in complexes with Kap1 might be present as lower-molecular-weight complexes, e.g., monomeric or dimeric complexes. Our observation that Kap1 is not enriched at the viral ori or in MCPyV genomes in general is supported by the finding that Kap1 is not recruited to LT replication centers in immunofluorescence stainings performed in primary cells transduced with replication-competent MCPyV genomes or infected with MCPyV.

In contrast, although we have observed reduced Kap1 binding at cellular promoter regions of the zinc finger proteins ZNF180 and ZNF274 upon expression of the MCPyV ER, our transcriptome analysis did not show significant overlapping of DEGs in cells devoid of Kap1 or in cells overexpressing LT (Fig. 8) such as would be expected if LT were to generally interfere with Kap1 recruitment to cellular promoters.

More recently, phosphorylation of Kap1 serine 824 was shown to represent an important posttranslational modification in regulating Kap1 function, with phosphorylation at this site resulting in an abrogation of SETDB1 binding and subsequent derepression of Kap1 targets. Rauwel and colleagues demonstrated that phosphorylation in CD34^+^ cells at that site represents a switch factor for the latent and lytic cycles of human-pathogenic CMV (hCMV) (31). Interestingly, that study also demonstrated that hCMV can be reactivated by activation of ATM.

Our findings indicate that MCPyV DNA replication results in the ATM-dependent phosphorylation of Kap1 S824 and in subsequent cell cycle arrest in primary nHDF cells. Interestingly, our experiments demonstrated that ATM-mediated Kap1 phosphorylation is a critical step for virus replication. Treatment of cells with an ATM inhibitor restored MCPyV replication in nHDF cells. We show that phosphorylation of Kap1 S824 is dependent on viral DNA replication, since a replication-defective viral mutant, MCPyV (K331A), which expresses LT at nearly wt levels at early time points, does not induce pKap S824. We also included a MCPyV mutant in which we replaced MCPyV LT S816A, representing a phosphorylation site in LT previously described to be important for restriction of cell proliferation and cell cycle arrest (38). This serine residue has been shown to be phosphorylated by ATM, and an ectopically expressed LT S816A mutant was reported to show a less severe form of restriction of C33A cells. In contrast, the S816A mutant behaved similarly to the wt virus in our experiments. This fact might be explained by the different cellular background and experimental system used in our study: we mutated LT in the context of the virus, and our experiments were performed in the presence of sT and other proteins of the early region.

In line with our hypothesis that DNA damage induced by MCPyV DNA replication restricts cell proliferation, we found that ectopic expression of T-Ag or ER in primary cells did not result in pKap S824 or in induction of p21 transcription. However, we observed that experiments performed with HEK293 cells, which contain parts of the adenovirus genome (46) and express adenoviral proteins E1A and E1B, resulted in pKap1S824 induction in response to MCPyV ER expression. A possible explanation for this observed difference between primary cells and HEK293 cells might be that HEK293 cells, due to their adenoviral protein expression pattern (and overlapping functions with respect to PyV early protein expression), are already preactivated with regard to DNA damage response and induction of Kap1 phosphorylation. Interestingly, our results indicate that nHDF cells harboring replicating MCPyV genomes arrest in G_2_ and undergo senescence, which we demonstrated by detection of increased levels of p21 transcript and β-galactosidase staining and an increase of in the number of transcripts coding for cytokines and growth factors known to contribute to a senescence phenotype-associated protein (SASP) complex (40, 42, 43).

Previously, human dermal fibroblasts were proposed as primary target cells of MCPyV infection. However, our results indicate that primary human dermal fibroblasts transfected with religated MCPyV genome undergo senescence. In line with efficient restriction of viral replication in these cells, we did not observe an increase of the levels of MCPyV-positive cells over time and no infectious virus was observed in the supernatant of the cultures (see Fig. S1D in the supplemental material). We hypothesize that the senescence phenotype in nHDF cells represents an efficient host defense mechanism against viral replication. At present, we do not know what the functional role of the observed interaction between sT/LT and Kap1 is. We suspect that it might serve to counteract the activity mediated by this pathway, but, if so, it evidently failed to rescue viral replication in our *in vitro* system. While we infected or transfected commercial nHDF cells from a single donor, it seems possible that this cell type can support MCPyV infection *in vivo* or in the previously described *ex vivo* model (14), due, for example, to the presence of paracrine factors provided by other cell types.

## MATERIALS AND METHODS

### Cell culture.

HEK293 (46), H1299 (47), and HeLa SUMO1/2 and HeLa Par cells (39) were grown as monolayer cultures in Dulbecco’s modified Eagle’s medium (DMEM) supplemented with 10% fetal bovine serum (FBS) and 5% penicillin/streptomycin. PFSK-1 cells (ATCC; CRL-2060) were grown in RPMI medium supplemented with 10% FBS,5% penicillin/streptomycin, and l-glutamine. nHDF cells (Lonza) were cultured in FGM-2 medium (PromoCell) supplemented with the corresponding BulletKit.

### Plasmids and transfection methods.

MCVSyn (MCPyV Syn), pCMV2B-ER, and peYFP-N1-ER have been described previously (15). MCPyV mutants K331A and S816A, pYFP-OBD-stop(Y530), and pYFP-Zn-stop(Y429) were generated by site-directed mutagenesis. Sequences of primers used in site-directed mutagenesis and cloning strategies for pYFP-trunc-LT (MCCL-12 [48]) and pCR2.1-MCPyV-NCCR are listed in Table S1 in the supplemental material.

10.1128/mBio.00142-20.8 TABLE S1 Oligonucleotide sequences. Download Table S1, PDF file, 0.01 MB. Copyright © 2020 Siebels et al. 2020 Siebels et al. This content is distributed under the terms of the Creative Commons Attribution 4.0 International license.

10.1128/mBio.00142-20.9 TABLE S2 Ingenuity pathway analysis. All differentially regulated genes correspond to comparisons 1 to 6. Download Table S2, PDF file, 0.01 MB. Copyright © 2020 Siebels et al. 2020 Siebels et al. This content is distributed under the terms of the Creative Commons Attribution 4.0 International license.

10.1128/mBio.00142-20.10 TABLE S3 List of genes associated with SASP. Using GeneCards (https://www.genecards.org/), 204 genes and 8 microRNAs were found to be associated with “senescence” and “SASP” (downloaded September 2018). Download Table S3, PDF file, 0.01 MB. Copyright © 2020 Siebels et al. 2020 Siebels et al. This content is distributed under the terms of the Creative Commons Attribution 4.0 International license.

Retroviral MCPyV sT or LT constructs were described before (49). GST-LT_1 − 258_, GST-LT_79 − 170_, and GST-LT_171 − 254_ have been published before (20). pcDNA3.1-FLAG-Kap1, pcDNA3.1-FLAG-Kap1ΔRBCC, pcDNA3.1-FLAG-Kap1ΔPB, and pcDNA3.1-FLAG-Kap1ΔRBCC/PB were kindly provided by P. Farnham (37). LeGo-FLAG-Kap1 and LeGo-FLAG-Kap1ΔRBCC were generated by cloning the FLAG-Kap1 inserts from the corresponding pcDNA3.1 vectors in LeGo-iC2.

HEK293, H1299, and HeLa cells were transfected with polyethylenimine (PEI; Polysciences, Inc.), nHDF cells were transfected using electroporation (Neon transfection system, 1 pulse, 1,700 V, 20 ms). PFSK-1 cells were transfected with X-tremeGENE (Roche).

### Immunofluorescence staining.

A total of 4 × 10^4^ nHDF cells were seeded on gelatin-coated coverslips and analyzed by immunofluorescence (36) using the following antibodies: anti-MCPyV LT (Cm2B4; Santa Cruz) (1:500), anti-Kap1 (catalog no. 10483; Abcam) (1:1,000), anti-pKap1 S824 (catalog no. 70369; Abcam) (1:1,000), anti-mouse Alexa Fluor 488 (Life Technologies; catalog no. A-11001), anti-mouse Cy5 (A10523), and anti-rabbit Alexa Fluor 555 (A-21428). Staining was analyzed on a Nikon spinning-disk confocal microscope with a 20× nonconfocal lens objective or a 100× confocal lens objective. Quantification of phosphorylation intensities was performed by applying the Fiji software package and ImageJ, evaluating 100 cells each.

### MCPyV replication assay.

*In vitro* replication assays were performed as previously described (15, 16, 35). For nHDF cells, 1 × 10^6^ cells were transfected with 2 μg MCVSyn or pUC18 plasmid (mock) using electroporation. Cells were resuspended in 100 μl buffer R and mixed with DNA (in double-distilled water [ddH_2_O]), and 100-μl Golden tips were used (1 pulse at 1,700 V for 20 ms). Cells were immediately transferred into 1 ml FGM-2 medium (Promo Cell) without antibiotics.

At 2, 4, or 8 days p.t., genomic DNA was isolated to determine MCPyV genome copy numbers (16, 36).

### MCPyV infection.

MCPyV infectious particles were produced following a protocol published recently (50). Infection of nHDF cells was performed as described previously (14).

### Senescence-associated β-galactosidase staining.

Early passages (P2 to P7) of nHDF cells were transfected with MCPyV genome by electroporation. β-Galactosidase staining was combined with an immunofluorescence assay (IFA), and the corresponding experiments were performed as previously described (51).

### Gene expression analysis by reverse transcription-quantitative PCR (RT-qPCR).

RNA was isolated by the use of RNA Bee (Amsbio) followed by DNase I digestion (Invitrogen). A 1-μg volume of RNA was used for random cDNA synthesis (Superscript III; Invitrogen).

### Transcriptome analysis.

Library preparation was carried out using a NEBNext Ultra RNA library preparation kit (Illumina) and 1 μg RNA. Libraries were sequenced on an Illumina HiSeq 2500 platform (SR50). Reads were mapped and counted on the hg38 human genome using Star v2.5 (52). DEGs were identified using DEseq2 (53). Differentially regulated genes were selected by calculation of a false-discovery rate below 0.1 (FDR < 0.1) and log_2_ fold change values greater than or equal to 1 and less than or equal to −1. Gene Ontology was performed using DAVID (54, 55) and Ingenuity pathway analysis (Qiagen).

### Western blotting.

Cells were washed in Tris-buffered saline (TBS), cooled on dry ice, and resuspended in radioimmunoprecipitation assay (RIPA) buffer (50 mM Tris-HCl [pH 7.5], 150 mM NaCl, 1% [vol/vol] NP 40, 0.5% [wt/vol] sodium deoxycholate, 5 mM EDTA, 0.1% [wt/vol] SDS, 1 mM NaF, 2 mM glycerolphosphate, 1 mM Na_3_VO_4_, cOmplete protease inhibitor cocktail, PhosSTOP [Sigma-Aldrich]). The antibodies used were as follows: anti-MCPyV LT (CM2B4l; Santa Cruz), anti-Kap1 (Abcam; catalog no. ab22553), anti-pKap1 S824 (Abcam; catalog no. ab70369), anti-actin (Santa Cruz; catalog no. sc-47778) and anti-sT-Ag (2T2).

### Coimmunoprecipitation and GST pulldown experiments.

Total cell extracts and Co-IPs were generated as described previously (17).

The SUMOylation status of Kap1 was determined in HeLa SUMO2 and HeLa parental cells as described previously (39). GST pulldown experiments were performed as described previously (20).

### Kap1 knockout cells and Kap1 rescue experiments.

pSpCas9(BB)-2A-GFP (PX458), Kap1 single guide RNA (sgRNA), or control sgRNA (56) was transfected into HEK293 or H1299 cells. At 2 days p.t., green fluorescent protein (GFP)-positive cells were sorted using a fluorescence-activated cell sorter (FACS) and dispensed by the single-cell deposit method. Kap1 expression was validated by Western blotting. Five and three positive clones of HEK293 and H1299 cells, respectively, were pooled. GFP-positive control cells were cultured in bulk. For rescue experiments, cells were transduced with lentiviral supernatant of plasmid LeGo-FLAG-Kap1 or plasmid LeGo-FLAG-Kap1ΔRBCC and bulk sorted for identification of mCherry-positive cells. Expression of Kap1 or mutant Kap1 was validated by Western blotting and IFA.

### DNA-protein interaction (DPI)–ELISA.

pNTAP-ER,(22) was transiently expressed in HEK293 Kap1 knockout and control cells. At 2 days p.t., cells were lysed in 10× RIPA buffer. The supernatant was supplemented with dilution buffer (50 mM Tris-HCl [pH 7.5], 150 mM NaCl, cOmplete protease inhibitor). Streptavidin Sepharose high-performance beads (GE Healthcare) were incubated with cell extracts overnight at 4°C and washed in a reaction mixture containing 50 mM Tris-HCl (pH 7.5), 150 mM NaCl, and 1% Triton X-100 followed by a mixture containing 50 mM Tris-HCl (pH 7.5), 500 mM NaCl, and 1% Triton X-100. LT was eluted (for 30 min at 4°C) in a reaction mixture containing 600 μl 20 mM Tris-HCl (pH 8.5), 150 mM NaCl, and 4 mM biotin. The DPI-ELISA was performed with 1 μg of purified LT protein as described before (22).

### ChIP-qPCR and ChIP-seq.

HEK293 cells (5 × 10^6^) were transfected with 10 μg pCMV2B-ER and 0.5 μg pCR2.1-MCPyV-NCCR or 10 μg pCMV2B-ER and 0.5 μg pMK-MCVSyn-stop. At 2 days p.t., ChIP was performed as described previously (57) using anti-LT antibody (Cm2B4) and anti-Kap1 antibody (ab10483; Abcam).

Sequencing libraries were prepared from 2 to 10 ng DNA using a NEXTflex Illumina ChIP-seq library prep kit (Bio Scientific) and were sequenced on an Illumina NextSeq (SR50) system. Sequencing reads were aligned to the human reference genome (hg19) and pCR2.1-MCPyV-NCCR using Bowtie version 1.2.2 (58). Sites with enriched Kap1 levels were detected by the use of MACS version 2.1.2 (59), and matched negative-control region sets were generated with EaSeq (60).

### EMSA.

A total of 5 × 10^6^ HEK293 Kap1 knockout and control cells were transiently transfected with 10 μg DNA. Nuclear extracts were prepared at 2 days p.t. (61, 62). Labeled probe was generated by annealing 100 pmol of each MCPyV-ori primer in 100 μl annealing buffer (10 mM Tris-HCl [pH 8.0], 1 mM EDTA, 50 mM NaCl) and by 5′ labeling with [γ-^32^P]ATP.

A 0.5-ng volume of MCPyV *ori* probe and 10 μg of nuclear extract were incubated for 20 min at room temperature in 20 μl binding buffer [30 mM Tris-HCl (pH 8.0), 10% glycerol, 5 mM AMP-PNP (Sigma), 25 ng/μl bovine serum albumin (BSA), 3 ng/μl sonicated sperm DNA, 0.5 ng/μl poly(dI-dC), 1 mM phenylmethylsulfonyl fluoride (PMSF)] (63). For supershifts, 0.5 μg Cm2B4 (Santa Cruz) was added (10 min at room temperature). Reactions were separated on a native polyacrylamide gel (4.5%), followed by autoradiography.

### Cell cycle analysis.

A total of 1 × 10^6^ nHDF cells were transfected with 2 μg MCPyV or control DNA using electroporation. At 2 and 4 days p.t., cells were fixed (70% ethanol), washed in phosphate-buffered saline (PBS), and resuspended in 200 μl FACS buffer (PBS, 1% FBS, 5 mM EDTA, 25 mM HEPES, 100 μg/ml RNase A) followed by propidium iodide staining and flow cytometry. FlowJo software and the Dean-Jett-Fox algorithm were used.

### Proliferation.

Cell proliferation was assessed by 3-(4,5-dimethyl-2-thiazolyl)-2,5-diphenyl-2H-tetrazolium bromide (MTT) assay or by automated cell counting (TC20 automated cell counter systems; Bio-Rad).

### Statistics.

Statistical analysis was performed in GraphPad Prism using *t* tests, including one-sample *t* tests, for samples following a normal distribution; the Mann-Whitney test and the Wilcoxon signed rank test were applied for nonparametric samples. For p21 transcripts, a mixed random intercept model was computed by the use of IBM SPSS software, taking the different starting point of p21 transcription into account. A two-tailed *P* value of <0.05 was considered significant.

### Data availability.

Transcriptome sequencing (RNA-Seq) data have been submitted to ENA under accession number PRJEB30502.
