# Supplementary figures and images for: Merkel Cell Polyomavirus DNA Replication Induces Senescence in Human Dermal Fibroblasts in a Kap1/Trim28-Dependent Manner
Source: mBio. 2020 Mar 10;11(2):e00142-20. doi: 10.1128/mBio.00142-20 (PMC7064754; doi:10.1128/mBio.00142-20)

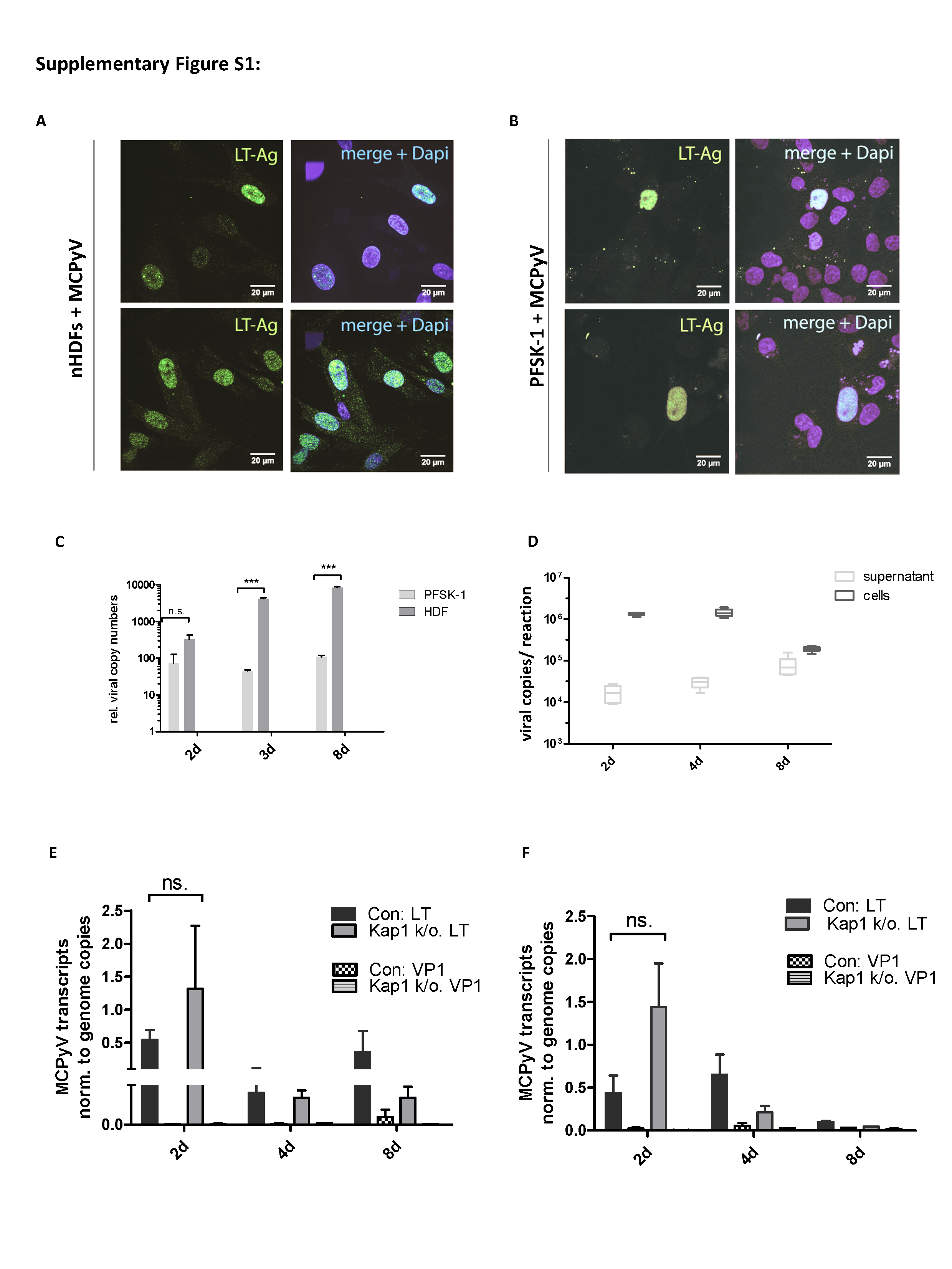

Supplement: FIG S1 [file mBio.00142-20-sf001.tif]

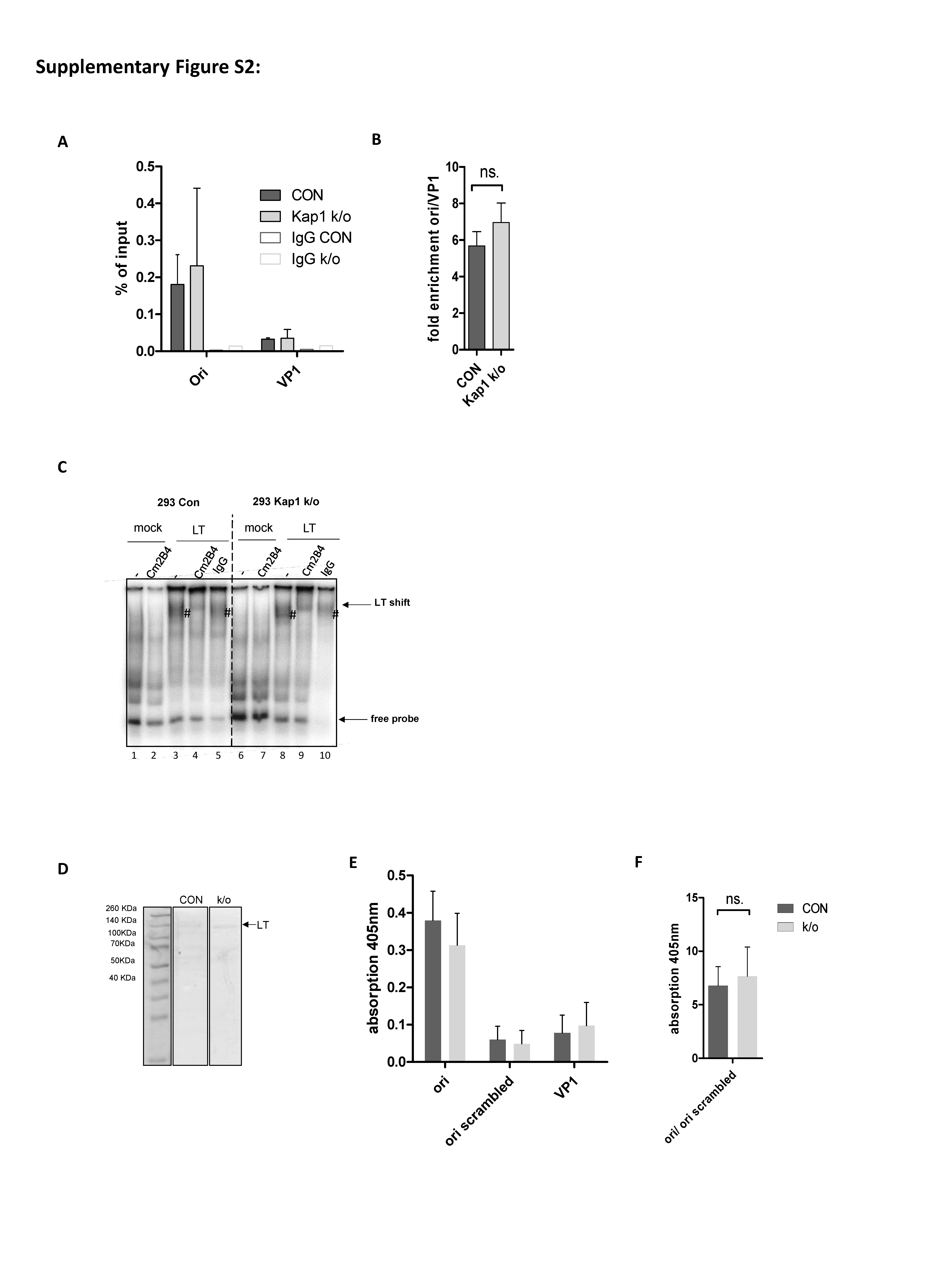

Supplement: FIG S2 [file mBio.00142-20-sf002.tif]

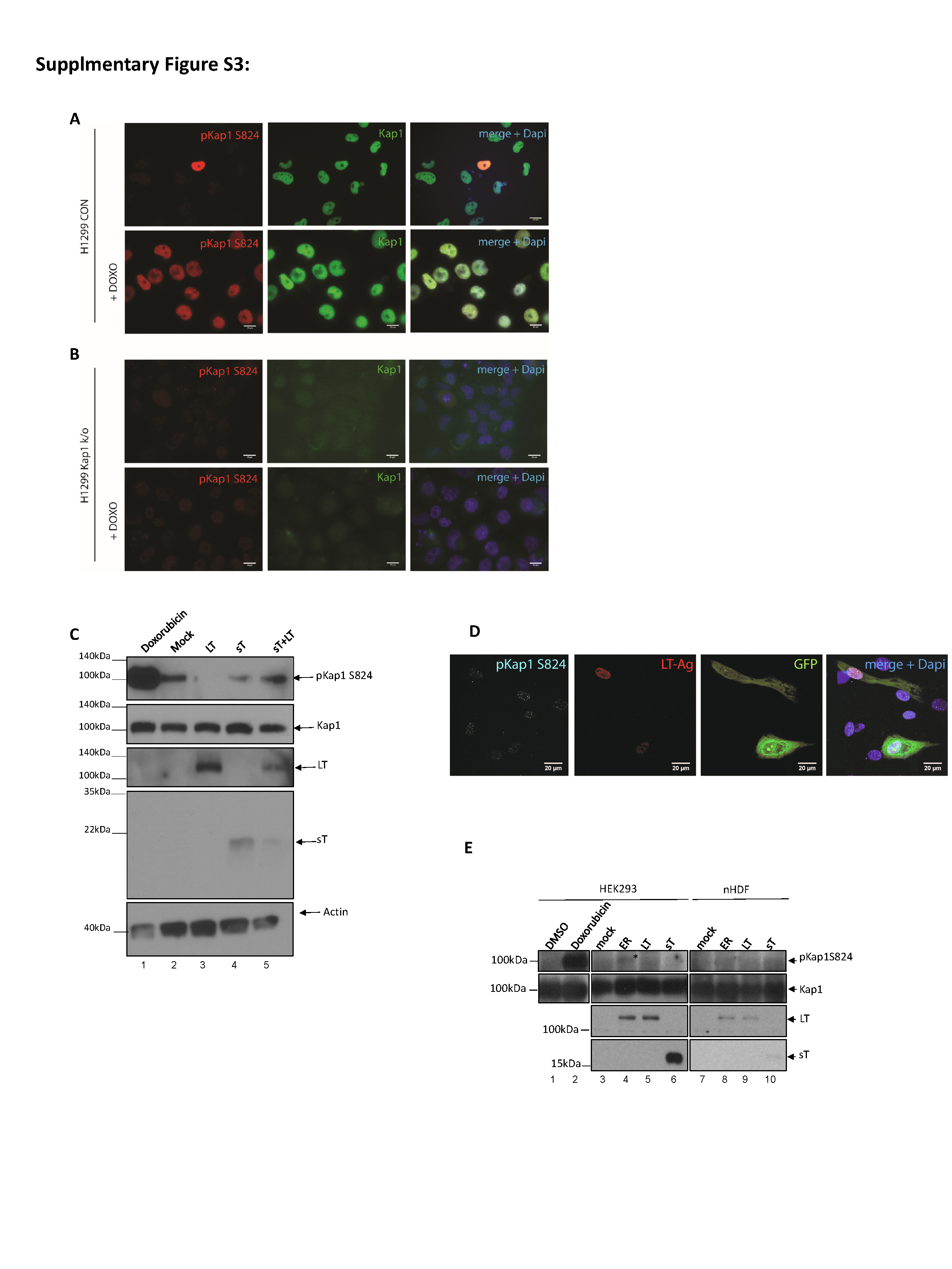

Supplement: FIG S3 [file mBio.00142-20-sf003.tif]

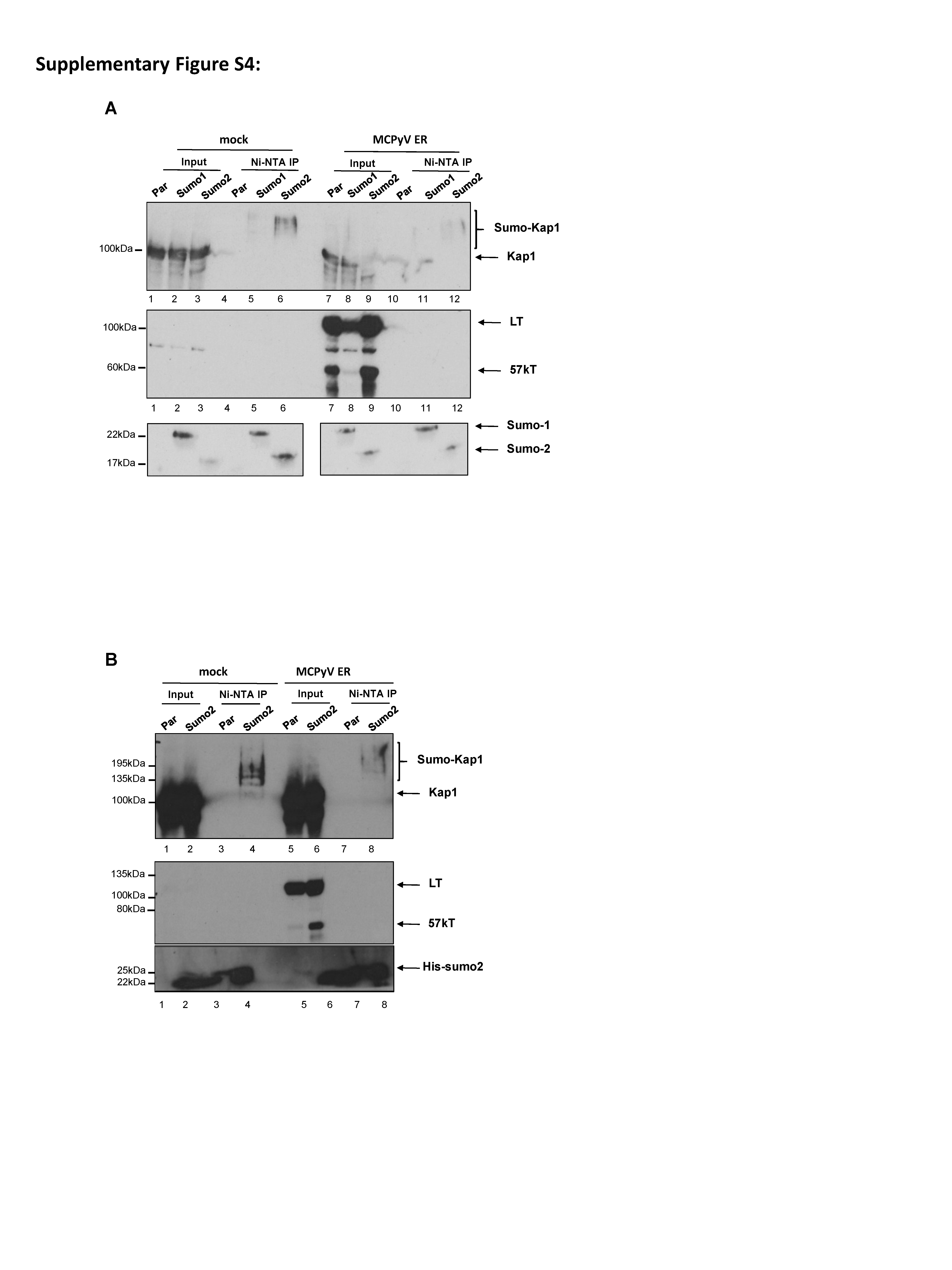

Supplement: FIG S4 [file mBio.00142-20-sf004.tif]

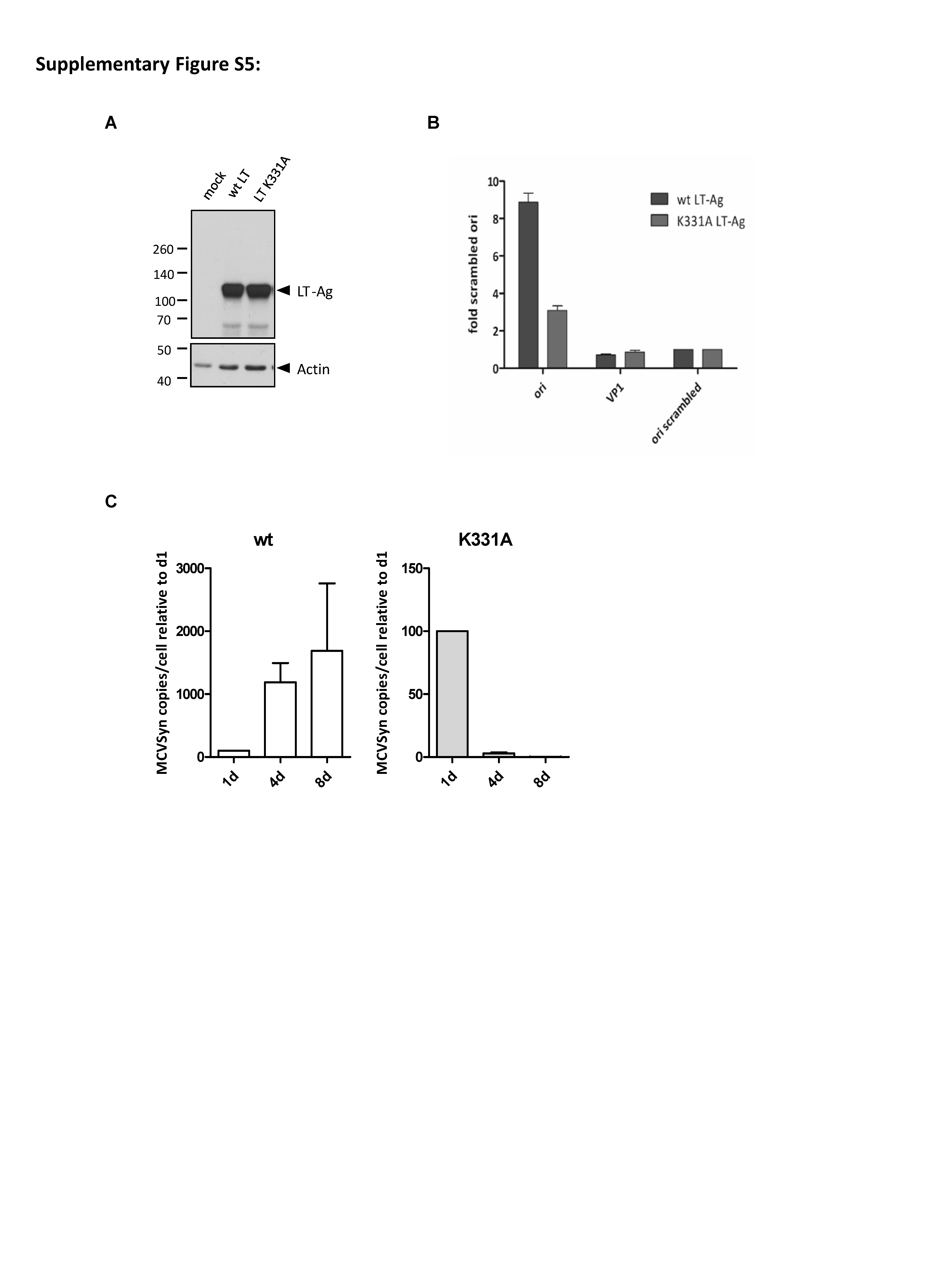

Supplement: FIG S5 [file mBio.00142-20-sf005.tif]

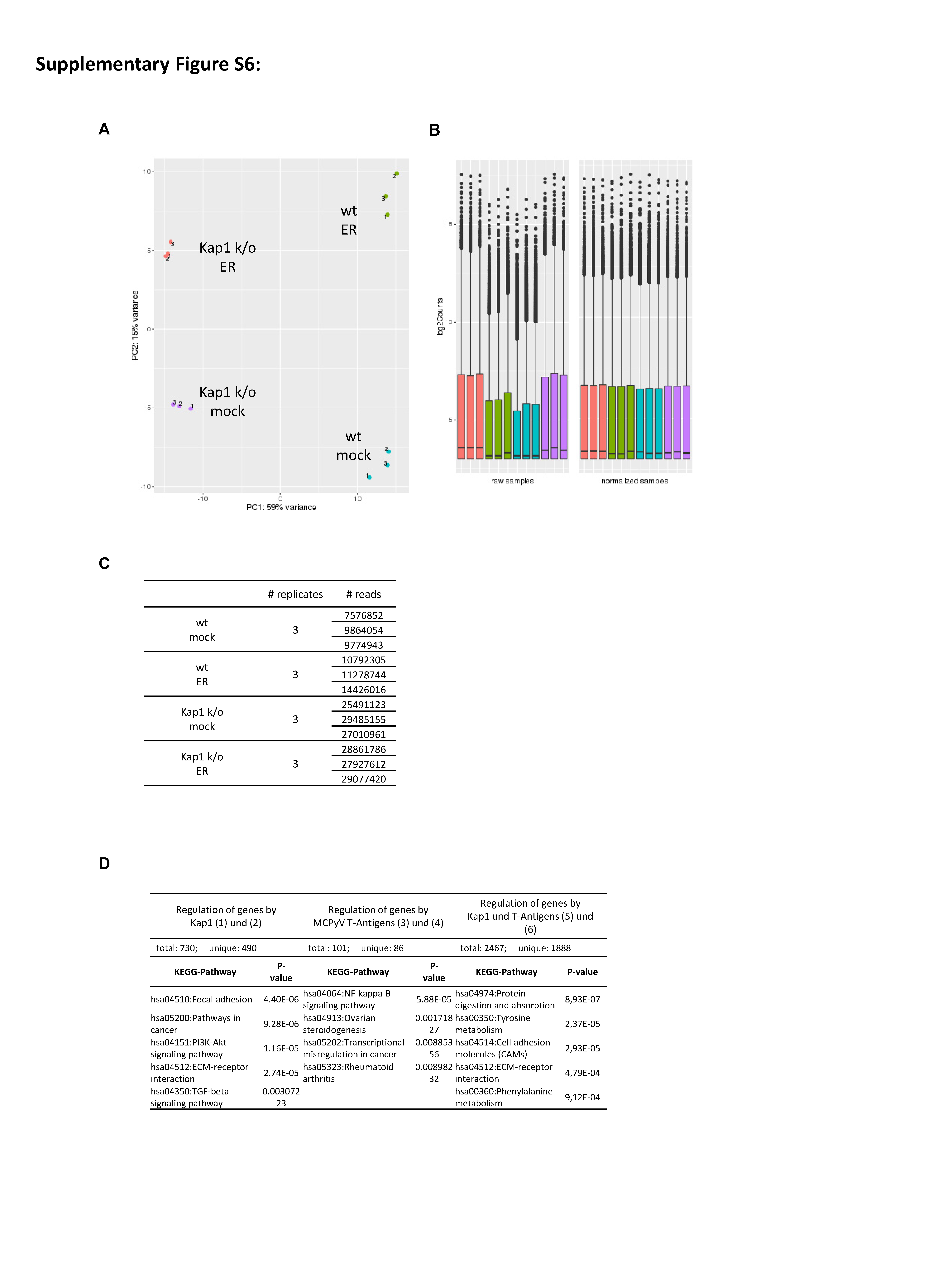

Supplement: FIG S6 [file mBio.00142-20-sf006.tif]

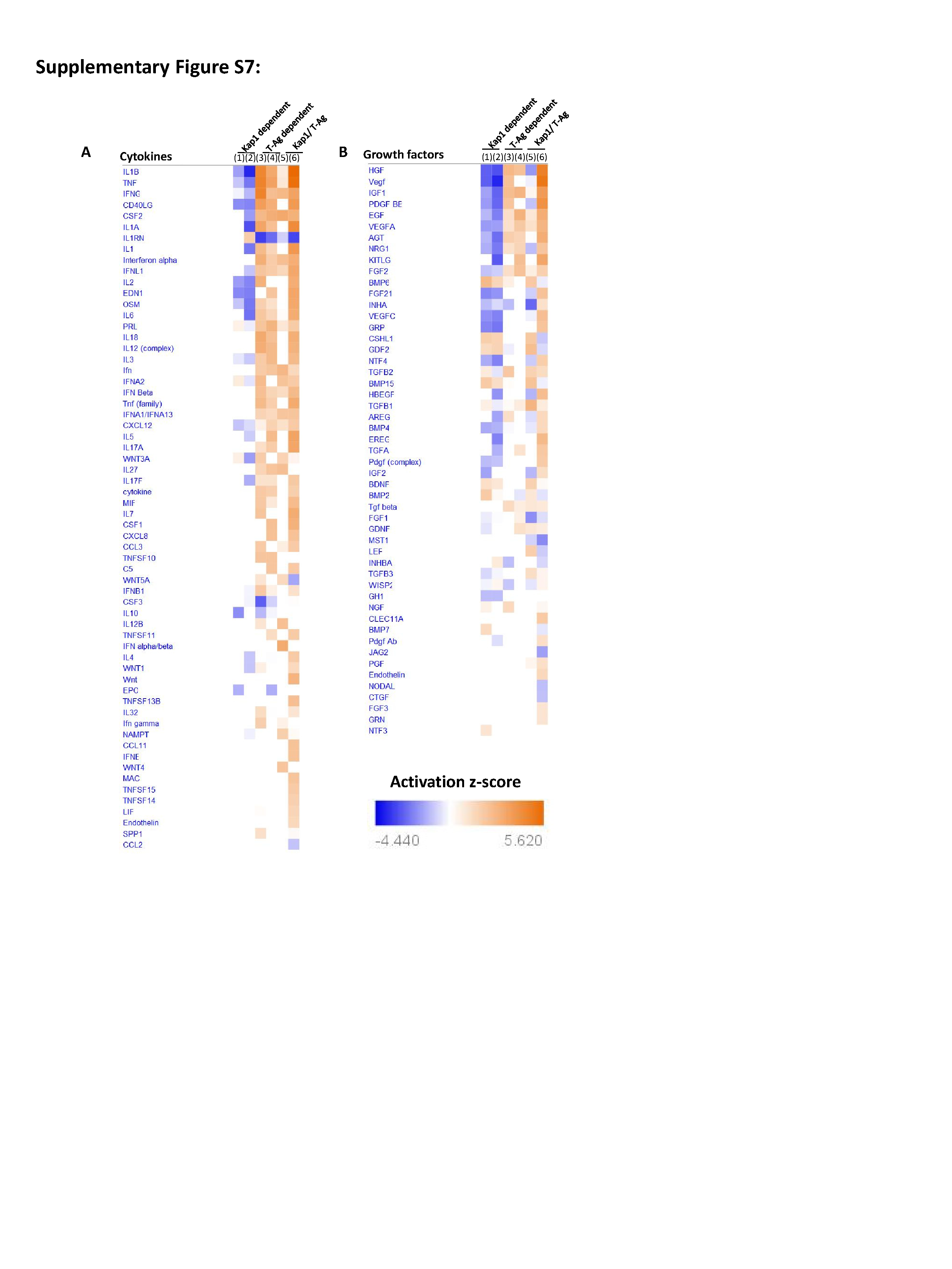

Supplement: FIG S7 [file mBio.00142-20-sf007.tif]
