# Supplementary material for: Merkel Cell Polyomavirus DNA Replication Induces Senescence in Human Dermal Fibroblasts in a Kap1/Trim28-Dependent Manner
Source: mBio. 2020 Mar 10;11(2):e00142-20. doi: 10.1128/mBio.00142-20 (PMC7064754; doi:10.1128/mBio.00142-20)
Supplement: TABLE S1 [file mBio.00142-20-st001.pdf]

Table S1: Oligonucleotides

Name	Sequence 5'→ 3'
QC-MCPyV-LT-K331A-fw	CTTAGCCATGCTGTATATAGTAATGCAACTGTTTCATGTTTTGCC
QC-MCPyV-LT-K331A-rev	GAATCGGTACGACATATATCATTACGTTGACAAAGTACAAAACGG
QC-MCPyV-S816A-fw	CCAAGATTCTGGTACTTTTgCTCAATAAAGGCATCTGCT
QC-MCPyV-S816A-fw	AGCAGATGCCTTTATTGAGcAAAAGTACCAGAATCTTGG
QC-OBD-stop(Y529)-fw	TAAATCTCAGTAAACCATTTGCCAAC
QC-OBD-stop(Y529)-rev	GATTCATAAAATAGCTTAGCATTAG
QC-Zn-stop(Y429)-fw	TGCTAAATTAAGAATTTCAAGAAAAAGAAAAAG
QC-Zn-stop(Y429)-rev	GTGGCTTATTCTCTTGCAG
RA-MCPyV-VP1-fw	AAAACACCCAAAAGGCAATG
RA-MCPyV-VP1-rev	GCAGAGACACTCTTGCCACA
GAPDH-fw	TGTGTCCCTCAATATGGTCCTGTC
GAPDH-rev	ATGGTGGTGAAGACGCCAGT
p21-fw	CTTGTACCCTTGTGCCTCGCT
p21-rev	CGGATTAGGGCTTCCTCTTGG
YWHAZ-fw	ACTTTTGGTACATTGTGGCTTCAA
YWHAZ-rev	CCGCCAGGACAAACCAGTAT
HPRT1-fw	TGACCTTGATTTATTTTGCATACC
HPRT1-rev	CGAGCAAGACGTTCAGTCCT
KAP1_sgRNA1fw	CACCgGGAGCGCTTTTCGCCGCCAG
KAP1_sgRNA1rev	AAACCTGGCGGCGAAAAGCGCTCCc
KAP1_sgRNA2fw	CACCgACTACAGGCCGAGTGCAAAC
KAP1_sgRNA2rev	AAACGTTTGCACTCGGCCTGTAGTc
CON_ sgRNAfw	CACCgGGGTACATCCGCTCGGAGG
CON_ sgRNArev	AAACCCTCCGAGCGGATGTACCCc
ori-fw	CTTGGCTGCCTAGGTGAC
ori-rev	TCTGCCCTTAGATACTGCCT
VP1-fw	CAGAGGGCTTTGGGTAAACAG
VP1-rev	CCAAAAGTGTCAGGCCAACC
ZNF180-fw	TGATGCACAATAAGTCGAGCA
ZNF180-rev	TGCAGTCAATGTGGGAAGTC
ZNF274-fw	GGAGAAATCCCATGAGGGTAA
ZNF274-rev	GGCTTTTGTGAGAATGTTTTCC

ACHE-fw	CTGGAATTACAGGGGTGAGC
ACHE-rev	GGGTCTTGCAGAGACAGAGG
MCPyV_ori1	AATTCAAGTTGGCAGAGGCTTGGGGCTCCTAGCCTCCGAGGCCTCTGGAAAAAAAAGAGAGAGGCCTCTGAGGCTTAAGAGGCTTAATTAG
MCPyV_ori2	AATTCTAATTAAGCCTCTTAAGCCTCAGAGGCCTCTCTCTTTTTTTTCCAGAGGCCTCGGAGGCTAGGAGCCCCAAGCCTCTGCCAACTTG
MCPyV_ori_bio1	TAATTAAGCCTCTTAAGCCTCAGAGGCCTCTCTCTTTTTTTTCCAGAGGCCTCGGAGGCTAGGAGCCCCAAGCCTCTGCC-BITEG
MCPyV_ori_unbio2	GGCAGAGGCTTGGGGCTCCTAGCCTCCGAGGCCTCTGGAAAAAAAAGAGAGAGGCCTCTGAGGCTTAAGAGGCTTAATTA
VP1_bio1	TGCATAGAGGGCCCACTCCATTCTCATCTAAAAGGACAGTAGTTAGAGTATTACTAAATTGAAGAACTGTAGGAGTCTGA - BIOTEG
VP1_unbio2	TCAGACTCCTACAGTTCTTCAATTTAGTAATACTCTAACTACTGTCCTTTTAGATGAGAATGGAGTGGGCCCTCTATGCA
Ori_scrm_bio1	GCTCTCTCGTCACACCTGCATCACCAGGAGACGTGACTGCTCACACCTTGGTATTCTCACACGTCGTATGGTCGTCTATG - BIOTEG
Ori_scrm_unbio2	CATAGACGACCATACGACGTGTGAGAATACCAAGGTGTGAGCAGTCACGTCTCCTGGTGATGCAGGTGTGACGAGAGAGC
MCPyV_unbio1	TAATTAAGCCTCTTAAGCCTCAGAGGCCTCTCTCTTTTTTTTCCAGAGGCCTCGGAGGCTAGGAGCCCCAAGCCTCTGCC
