# Supplementary material for: Merkel Cell Polyomavirus DNA Replication Induces Senescence in Human Dermal Fibroblasts in a Kap1/Trim28-Dependent Manner
Source: mBio. 2020 Mar 10;11(2):e00142-20. doi: 10.1128/mBio.00142-20 (PMC7064754; doi:10.1128/mBio.00142-20)
Supplement: TABLE S2 [file mBio.00142-20-st002.pdf]

© 2000-2018 QIAGEN. All rights reserved.

Upstream reg	kap1 k/o vs wt	kap1 k/o LT vs LT	LT vs wt	kap1 k/o LT vs kap1 k/o	kap1 k/o LT vs wt	LT vs kap1 k/o
NFkB (comple	-1.888850064	-3.604678218	4.2394593	3.762391356	1.444377181	4.705710038
IL1B	-1.688224691	-3.789868945	4.519979297	3.256190983	0.767027647	5.6203484
TNF	-0.997912829	-2.38401268	4.651589446	3.525915973	0.924498718	5.413583006
F2	-1.701594712	-3.372076502	3.850688093	2.907425456	0.893483778	5.035879274
tretinoin	-1.368711176	-2.570941138	3.944597897	3.046042715	1.411698261	5.214178973
HGF	-2.801965261	-3.061660651	2.680364163	2.350370444	-1.819830622	4.719602323
lipopolysacch	-1.395589853	-2.430576803	3.373870786	2.782111567	1.735672448	5.389992048
poly rI:rC-RN	N/A	-2.489560223	3.811591685	3.288180424	1.763207262	4.868089478
LY294002	N/A	2.116007813	-3.751015373	-3.480783351	-2.393260148	-4.440173247
IFNG	-0.218655681	-1.256348352	4.657135287	2.456667845	2.641616167	3.460015755
CD40LG	-2.013443998	-2.142018248	3.639340904	3.024926165	N/A	3.811479648
E. coli B5 lipo	N/A	-3.034505707	2.230526227	2.738335045	2.69916172	3.921547718
Vegf	-2.813830945	-3.804458698	2.334014174	N/A	-0.280388707	5.123267919
phorbol myris	-0.743639784	-1.488965125	3.873052226	3.116243152	1.121401778	3.769936167
U0126	0.552167959	2.010715026	-3.637116934	-2.525036258	-1.353652882	-3.949364162
HIF1A	-2.629151955	-3.259384724	2.718717023	1.05670222	-0.476010344	3.870770676
Alpha catenin	1.672822428	2.303902726	-2.945431154	-2.449489743	-0.523065781	-3.979366803
IGF1	-1.62624588	-2.733441297	2.498877835	2.774681072	0.38053806	3.735313044
CSF2	N/A	-1.768710404	2.632173949	3.051256547	3.249265725	2.95210684
P38 MAPK	-2.060149682	-2.609310239	3.124369412	1.974069607	-0.029380079	3.808173066
IRF1	2.384615385	N/A	3.061441371	2.167683173	3.229194035	2.71779984
F2R	-2.249363771	-2.807642989	2.392810166	2.392810166	0.194192777	3.485287128
PD98059	2.675751303	2.411936381	-2.136977829	-2.811024001	0.00511114	-3.47534363
decitabine	-0.729590316	-2.075207589	2.44360465	3.073630584	0.711764919	4.409290168
E. coli B4 lipo	N/A	N/A	3.180432496	2.900617387	3.605278294	3.739073239
TLR3	-2.04264872	-1.978795069	2.877418755	2.196096341	N/A	4.076747324
IL1A	N/A	-3.054097293	3.373798938	2.363560043	N/A	4.351516693
SMARCA4	-0.835889824	-1.796776615	3.381098951	N/A	2.075275988	4.867683855
Tlr	N/A	-1.979898987	2.585699048	2.207234949	2.59026422	3.360060173
SP1	1.800741251	1.244485457	2.37468006	1.959620423	4.278412276	1.012696383
salmonella mi	N/A	N/A	3.233519404	3.107594384	3.709755129	2.589202602
Salmonella er	N/A	-1.8543453	2.37163587	2.390457219	1.359757654	4.507133701
MYD88	N/A	N/A	2.87063264	2.845802444	3.643194078	3.071127808
RELA	-1.63783002	-1.39086977	2.183182864	2.749765932	0.783349864	3.548987981
ATF4	-3.090995423	-2.153041795	-1.714985851	N/A	-3.388579283	1.745627936
IL1RN	N/A	1.923356623	-3.357401583	-2.584125422	-0.818477059	-3.229846132
tributyrin	N/A	-2.562726589	2.156655464	2.42535625	0.551677284	4.140949823
PDGF BB	-1.743413064	-2.670910973	2.218525792	N/A	-1.053385521	4.137626113
thapsigargin	-3.103182834	-2.551226709	1.268846756	N/A	-1.900052535	2.861064695
EGF	-1.246448969	-2.24604454	1.242499649	2.898756235	0.91647232	3.062287718
ERK1/2	-1.858113389	-1.239163937	2.053178269	2.18616638	0.713948129	3.518850641
FOXA1	2.657480442	3.146077244	N/A	N/A	3.128626808	-2.6167471
TLR7	N/A	N/A	3.222141459	2.057114154	2.735444973	3.480150802
TICAM1	N/A	N/A	3.138811324	2.970442629	2.963237119	2.413254211
doxorubicin	-1.645139417	-1.800445561	2.594732842	2.172620473	-0.050888225	3.041145499
KLF3	1.520526225	2.777696227	N/A	N/A	4.152697672	-2.72939781
TCF7L2	-1.90252179	-2.560509004	3.433758535	N/A	N/A	3.23214696
EGLN	2.09219869	3.209725674	N/A	N/A	2.52152076	-3.042698495
5-azacytidine	0.858008345	N/A	2.749035389	1.963961012	3.202437592	1.980549328
bromodeoxyu	N/A	N/A	2.411764706	2.200431147	2.967301476	3.147573112
STAT1	N/A	0.226671074	2.783213273	2.424181521	3.648244917	1.617815771
ESR1	-2.268138375	-2.598911765	-0.220093167	-0.765364973	-3.312425924	1.495033976
AGT	-1.283557346	-2.534974848	1.837599502	1.617724247	0.07832997	3.071962351
IL1	N/A	-2.381683528	2.572745809	1.545496343	N/A	3.881265561
Interferon alpl	N/A	N/A	3.00037178	1.917092858	2.409269537	3.023512476
APP	-0.421790092	N/A	3.0571384	1.612821556	1.795485343	3.265780238
salirasib	2.167774924	3.16227766	N/A	N/A	1.53118206	-3.285714286
JAK1/2	N/A	N/A	3	2.236067977	2.98240454	1.897366596
F3	-2.449489743	-1.897366596	N/A	1.99174119	N/A	3.761904762
IKBKB	N/A	-1.631167117	2.463351409	2.392862329	0.37097344	3.171936083
IFNL1	N/A	-1.037571696	2.163657997	1.929157714	1.5	3.303335203
IL2	-1.739617733	-2.153566232	2.9386866	N/A	N/A	3.055933622
EDN1	-2.097902878	-2.244969717	N/A	2.181579467	N/A	3.337831393
stallimycin	N/A	N/A	2.411764706	2.200431147	2.064187386	3.147573112
mitomycin C	-2.195308987	-2.396153312	0.21821789	N/A	-3.088251615	1.858192682
peptidoglycan	-1.947580113	N/A	2.337996177	1.949613781	N/A	3.509891358
TLR4	N/A	N/A	3.085495612	2.330367477	2.626578937	1.654581157
OSM	-1.021605841	-2.385384248	1.783470614	1.181113388	N/A	3.257580365
cyclic AMP	-0.604508271	-2.089615848	1.925625253	1.851739181	N/A	3.119513828
IL6	N/A	-2.487268879	2.099025733	1.563046808	0.158355748	3.104264867
RAF1	-2.293368754	-2.106063774	0.673587788	N/A	-0.733114955	3.565886627
DDX58	N/A	0.355618138	2.173971906	1.726225639	3.215095959	1.784753389
INSIG1	2.137667012	3.147573112	N/A	N/A	N/A	-3.896637497
Pka	-1.393261092	-2.253789254	N/A	N/A	-2.195287584	3.27416856
CTNNB1	-0.363224984	-2.034615017	2.292428988	N/A	1.293986867	3.053252726
Nr1h	N/A	N/A	-2.37607738	-2.389564306	-3.353297325	-0.725868337
KITLG	N/A	-2.904665541	N/A	2.392810166	N/A	3.36816093
TLR9	N/A	N/A	2.766097192	2.229710186	N/A	3.602325271
JUN	-0.636882685	-1.984443587	2.928813475	N/A	N/A	3.0432314
IL18	N/A	N/A	3.213064979	2.360200577	N/A	3.008932677
IL12 (complex	N/A	N/A	3.079885138	2.591772382	N/A	2.902957128
AHR	-0.256464837	1.71704535	-3.267956704	N/A	-1.147849249	-1.951424446
calphostin C	N/A	2.590369655	-2.20527146	N/A	N/A	-3.537343223
REL	N/A	N/A	3.049788484	2.200431147	N/A	3.063942307
Nfat (family)	N/A	-2.156395834	2.584935654	N/A	N/A	3.3194386
5-O-mycolyl-b	N/A	N/A	2.529822128	2.236067977	N/A	3.16227766
mycophenolic	N/A	N/A	2.121320344	1.80838886	N/A	3.887560546
SMARCB1	-1.068900648	-2.489503657	N/A	N/A	N/A	4.041451884
Tnf (family)	N/A	N/A	2.570150072	1.843637477	N/A	3.164044301
CpG oligonuc	N/A	-2.376354103	N/A	1.982481414	N/A	3.210329871
miR-155-5p (r	1.528486982	N/A	-1.803400192	N/A	0.800261685	-3.055854403
STAT4	-1.553797192	-2.306226641	N/A	N/A	0.032686023	3.285202648
CD40	N/A	N/A	3.008290285	2.174277302	N/A	1.836637872
IL5	N/A	-0.893437984	N/A	2.562848432	N/A	3.399549828
Ap1	N/A	-1.948362155	1.162398627	N/A	N/A	3.607618072
FN1	-0.712669833	N/A	N/A	2.09554797	0.763951886	3.070508904
NKX2-3	-1.297074953	1.14032E-16	-3.050851079	N/A	-1.855949774	0.363696484
IL17A	N/A	N/A	1.197133094	1.911153097	N/A	3.352321984
COL18A1	N/A	N/A	-1.091089451	-2.218800785	N/A	-3.059874484
CEBPA	N/A	-1.43653985	1.805889842	N/A	N/A	3.105366681
PRKCA	N/A	N/A	2.76352797	N/A	N/A	3.256144478
CHUK	-0.622572806	-1.059323516	N/A	1.176826846	N/A	3.123914912
CEBPB	-1.774005535	N/A	N/A	0.737190663	N/A	3.235918857
IL7	N/A	N/A	2.190251766	N/A	N/A	3.101246908
F2RL1	N/A	-2.114776409	N/A	N/A	N/A	3.055529923
GAST	N/A	-1.880331129	N/A	N/A	N/A	3.038037544
IFN alpha/beta	N/A	N/A	N/A	N/A	3.156044491	N/A

© 2000-2018 QIAGEN. All rights reserved.

Upstream reg	**kap1 k/o vs wt**	**kap1 k/o LT vs LT**	**LT vs wt**	**kap1 k/o LT vs kap1 k/o**	**kap1 k/o LT vs wt**	**LT vs kap1 k/o**
NFkB (comple	-1.888850064	-3.604678218	4.2394593	3.762391356	1.444377181	4.705710038
IL1B	-1.688224691	-3.789868945	4.519979297	3.256190983	0.767027647	5.6203484
TNF	-0.997912829	-2.38401268	4.651589446	3.525915973	0.924498718	5.413583006
F2	-1.701594712	-3.372076502	3.850688093	2.907425456	0.893483778	5.035879274
tretinoin	-1.368711176	-2.570941138	3.944597897	3.046042715	1.411698261	5.214178973
HGF	-2.801965261	-3.061660651	2.680364163	2.350370444	-1.819830622	4.719602323
lipopolysacch	-1.395589853	-2.430576803	3.373870786	2.782111567	1.735672448	5.389992048
poly rI:rC-RN/	N/A	-2.489560223	3.811591685	3.288180424	1.763207262	4.868089478
LY294002	N/A	2.116007813	-3.751015373	-3.480783351	-2.393260148	-4.440173247
IFNG	-0.218655681	-1.256348352	4.657135287	2.456667845	2.641616167	3.460015755
CD40LG	-2.013443998	-2.142018248	3.639340904	3.024926165	N/A	3.811479648
E. coli B5 lipo	N/A	-3.034505707	2.230526227	2.738335045	2.69916172	3.921547718
Vegf	-2.813830945	-3.804458698	2.334014174	N/A	-0.280388707	5.123267919
phorbol myris	-0.743639784	-1.488965125	3.873052226	3.116243152	1.121401778	3.769936167
U0126	0.552167959	2.010715026	-3.637116934	-2.525036258	-1.353652882	-3.949364162
HIF1A	-2.629151955	-3.259384724	2.718717023	1.05670222	-0.476010344	3.870770676
Alpha catenin	1.672822428	2.303902726	-2.945431154	-2.449489743	-0.523065781	-3.979366803
IGF1	-1.62624588	-2.733441297	2.498877835	2.774681072	0.38053806	3.735313044
CSF2	N/A	-1.768710404	2.632173949	3.051256547	3.249265725	2.95210684
P38 MAPK	-2.060149682	-2.609310239	3.124369412	1.974069607	-0.029380079	3.808173066
IRF1	2.384615385	N/A	3.061441371	2.167683173	3.229194035	2.71779984
F2R	-2.249363771	-2.807642989	2.392810166	2.392810166	0.194192777	3.485287128
PD98059	2.675751303	2.411936381	-2.136977829	-2.811024001	0.00511114	-3.47534363
decitabine	-0.729590316	-2.075207589	2.44360465	3.073630584	0.711764919	4.409290168
E. coli B4 lipo	N/A	N/A	3.180432496	2.900617387	3.605278294	3.739073239
TLR3	-2.04264872	-1.978795069	2.877418755	2.196096341	N/A	4.076747324
IL1A	N/A	-3.054097293	3.373798938	2.363560043	N/A	4.351516693
SMARCA4	-0.835889824	-1.796776615	3.381098951	N/A	2.075275988	4.867683855
Tlr	N/A	-1.979898987	2.585699048	2.207234949	2.59026422	3.360060173
SP1	1.800741251	1.244485457	2.37468006	1.959620423	4.278412276	1.012696383
salmonella m	N/A	N/A	3.233519404	3.107594384	3.709755129	2.589202602
Salmonella e	N/A	-1.8543453	2.37163587	2.390457219	1.359757654	4.507133701
MYD88	N/A	N/A	2.87063264	2.845802444	3.643194078	3.071127808
RELA	-1.63783002	-1.39086977	2.183182864	2.749765932	0.783349864	3.548987981
ATF4	-3.090995423	-2.153041795	-1.714985851	N/A	-3.388579283	1.745627936
IL1RN	N/A	1.923356623	-3.357401583	-2.584125422	-0.818477059	-3.229846132
tributyrin	N/A	-2.562726589	2.156655464	2.42535625	0.551677284	4.140949823
PDGF BB	-1.743413064	-2.670910973	2.218525792	N/A	-1.053385521	4.137626113
thapsigargin	-3.103182834	-2.551226709	1.268846756	N/A	-1.900052535	2.861064695
EGF	-1.246448969	-2.24604454	1.242499649	2.898756235	0.91647232	3.062287718
ERK1/2	-1.858113389	-1.239163937	2.053178269	2.18616638	0.713948129	3.518850641
FOXA1	2.657480442	3.146077244	N/A	N/A	3.128626808	-2.6167471
TLR7	N/A	N/A	3.222141459	2.057114154	2.735444973	3.480150802
TICAM1	N/A	N/A	3.138811324	2.970442629	2.963237119	2.413254211
doxorubicin	-1.645139417	-1.800445561	2.594732842	2.172620473	-0.050888225	3.041145499
KLF3	1.520526225	2.777696227	N/A	N/A	4.152697672	-2.72939781
TCF7L2	-1.90252179	-2.560509004	3.433758535	N/A	N/A	3.23214696
EGLN	2.09219869	3.209725674	N/A	N/A	2.52152076	-3.042698495
5-azacytidine	0.858008345	N/A	2.749035389	1.963961012	3.202437592	1.980549328
bromodeoxyu	N/A	N/A	2.411764706	2.200431147	2.967301476	3.147573112
STAT1	N/A	0.226671074	2.783213273	2.424181521	3.648244917	1.617815771
ESR1	-2.268138375	-2.598911765	-0.220093167	-0.765364973	-3.312425924	1.495033976
AGT	-1.283557346	-2.534974848	1.837599502	1.617724247	0.07832997	3.071962351
IL1	N/A	-2.381683528	2.572745809	1.545496343	N/A	3.881265561
Interferon alp	N/A	N/A	3.00037178	1.917092858	2.409269537	3.023512476
APP	-0.421790092	N/A	3.0571384	1.612821556	1.795485343	3.265780238
salirasib	2.167774924	3.16227766	N/A	N/A	1.53118206	-3.285714286
JAK1/2	N/A	N/A	3	2.236067977	2.98240454	1.897366596
F3	-2.449489743	-1.897366596	N/A	1.99174119	N/A	3.761904762
IKBKB	N/A	-1.631167117	2.463351409	2.392862329	0.37097344	3.171936083
IFNL1	N/A	-1.037571696	2.163657997	1.929157714	1.5	3.303335203
IL2	-1.739617733	-2.153566232	2.9386866	N/A	N/A	3.055933622
EDN1	-2.097902878	-2.244969717	N/A	2.181579467	N/A	3.337831393
stallimycin	N/A	N/A	2.411764706	2.200431147	2.064187386	3.147573112
mitomycin C	-2.195308987	-2.396153312	0.21821789	N/A	-3.088251615	1.858192682
peptidoglycar	-1.947580113	N/A	2.337996177	1.949613781	N/A	3.509891358
TLR4	N/A	N/A	3.085495612	2.330367477	2.626578937	1.654581157
OSM	-1.021605841	-2.385384248	1.783470614	1.181113388	N/A	3.257580365
cyclic AMP	-0.604508271	-2.089615848	1.925625253	1.851739181	N/A	3.119513828
IL6	N/A	-2.487268879	2.099025733	1.563046808	0.158355748	3.104264867
RAF1	-2.293368754	-2.106063774	0.673587788	N/A	-0.733114955	3.565886627
DDX58	N/A	0.355618138	2.173971906	1.726225639	3.215095959	1.784753389
INSIG1	2.137667012	3.147573112	N/A	N/A	N/A	-3.896637497
Pka	-1.393261092	-2.253789254	N/A	N/A	-2.195287584	3.27416856
CTNNB1	-0.363224984	-2.034615017	2.292428988	N/A	1.293986867	3.053252726
Nr1h	N/A	N/A	-2.37607738	-2.389564306	-3.353297325	-0.725868337
KITLG	N/A	-2.904665541	N/A	2.392810166	N/A	3.36816093
TLR9	N/A	N/A	2.766097192	2.229710186	N/A	3.602325271
JUN	-0.636882685	-1.984443587	2.928813475	N/A	N/A	3.0432314
IL18	N/A	N/A	3.213064979	2.360200577	N/A	3.008932677
IL12 (comple:	N/A	N/A	3.079885138	2.591772382	N/A	2.902957128
AHR	-0.256464837	1.71704535	-3.267956704	N/A	-1.147849249	-1.951424446
calphostin C	N/A	2.590369655	-2.20527146	N/A	N/A	-3.537343223
REL	N/A	N/A	3.049788484	2.200431147	N/A	3.063942307
Nfat (family)	N/A	-2.156395834	2.584935654	N/A	N/A	3.3194386
5-O-mycolyl-l	N/A	N/A	2.529822128	2.236067977	N/A	3.16227766
mycophenolic	N/A	N/A	2.121320344	1.80838886	N/A	3.887560546
SMARCB1	-1.068900648	-2.489503657	N/A	N/A	N/A	4.041451884
Tnf (family)	N/A	N/A	2.570150072	1.843637477	N/A	3.164044301
CpG oligonuc	N/A	-2.376354103	N/A	1.982481414	N/A	3.210329871
miR-155-5p (	1.528486982	N/A	-1.803400192	N/A	0.800261685	-3.055854403
STAT4	-1.553797192	-2.306226641	N/A	N/A	0.032686023	3.285202648
CD40	N/A	N/A	3.008290285	2.174277302	N/A	1.836637872
IL5	N/A	-0.893437984	N/A	2.562848432	N/A	3.399549828
Ap1	N/A	-1.948362155	1.162398627	N/A	N/A	3.607618072
FN1	-0.712669833	N/A	N/A	2.09554797	0.763951886	3.070508904
NKX2-3	-1.297074953	1.14032E-16	-3.050851079	N/A	-1.855949774	0.363696484
IL17A	N/A	N/A	1.197133094	1.911153097	N/A	3.352321984
COL18A1	N/A	N/A	-1.091089451	-2.218800785	N/A	-3.059874484
CEBPA	N/A	-1.43653985	1.805889842	N/A	N/A	3.105366681
PRKCA	N/A	N/A	2.76352797	N/A	N/A	3.256144478
CHUK	-0.622572806	-1.059323516	N/A	1.176826846	N/A	3.123914912
CEBPB	-1.774005535	N/A	N/A	0.737190663	N/A	3.235918857
IL7	N/A	N/A	2.190251766	N/A	N/A	3.101246908
F2RL1	N/A	-2.114776409	N/A	N/A	N/A	3.055529923
GAST	N/A	-1.880331129	N/A	N/A	N/A	3.038037544
IFN alpha/bet	N/A	N/A	N/A	N/A	3.156044491	N/A
