# Supplementary material for: Merkel Cell Polyomavirus DNA Replication Induces Senescence in Human Dermal Fibroblasts in a Kap1/Trim28-Dependent Manner
Source: mBio. 2020 Mar 10;11(2):e00142-20. doi: 10.1128/mBio.00142-20 (PMC7064754; doi:10.1128/mBio.00142-20)
Supplement: TABLE S3 [file mBio.00142-20-st003.pdf]

Download 05.07.2010 keyword: #SASP AND #senescence

0	Symbol	Description	Category	GIFtS	GC id
1	CDKN2A	Cyclin Dependent Kinase Inhibitor 2A	Protein Coding	59	GC09M021957
2	TP53	Tumor Protein P53	Protein Coding	65	GC17M007661
3	CDKN1A	Cyclin Dependent Kinase Inhibitor 1A	Protein Coding	61	GC06P040154
4	SIRT1	Sirtuin 1	Protein Coding	59	GC10P067884
5	RB1	RB Transcriptional Corepressor 1	Protein Coding	59	GC13P048303
6	CXCL8	C-X-C Motif Chemokine Ligand 8	Protein Coding	47	GC04P073740
7	IL6	Interleukin 6	Protein Coding	59	GC07P022765
8	MAPK14	Mitogen-Activated Protein Kinase 14	Protein Coding	61	GC06P040140
9	CDKN1B	Cyclin Dependent Kinase Inhibitor 1B	Protein Coding	59	GC12P012716
10	NFKB1	Nuclear Factor Kappa B Subunit 1	Protein Coding	63	GC04P102501
11	CDK6	Cyclin Dependent Kinase 6	Protein Coding	63	GC07M092604
12	ATM	ATM Serine/Threonine Kinase	Protein Coding	65	GC11P108127
13	IL1A	Interleukin 1 Alpha	Protein Coding	54	GC02M112773
14	BMI1	BMI1 Proto-Oncogene, Polycomb Ring Finger	Protein Coding	53	GC10P022326
15	CDK2	Cyclin Dependent Kinase 2	Protein Coding	63	GC12P055966
16	MTOR	Mechanistic Target Of Rapamycin Kinase	Protein Coding	63	GC01M011166
17	STAT3	Signal Transducer And Activator Of Transcription 3	Protein Coding	62	GC17M042313
18	MAPK1	Mitogen-Activated Protein Kinase 1	Protein Coding	63	GC22M021754
19	RELA	RELA Proto-Oncogene, NF-KB Subunit	Protein Coding	59	GC11M065671
20	CDK4	Cyclin Dependent Kinase 4	Protein Coding	67	GC12M057743
21	H2AFX	H2A Histone Family Member X	Protein Coding	54	GC11M119095
22	IGFBP7	Insulin Like Growth Factor Binding Protein 7	Protein Coding	55	GC04M057030
23	TNF	Tumor Necrosis Factor	Protein Coding	64	GC06P031673
24	CAV1	Caveolin 1	Protein Coding	59	GC07P116524
25	CCNA2	Cyclin A2	Protein Coding	55	GC04M121816
26	CDKN2B	Cyclin Dependent Kinase Inhibitor 2B	Protein Coding	56	GC09M021992
27	JUN	Jun Proto-Oncogene, AP-1 Transcription Factor Subunit	Protein Coding	60	GC01M058780
28	MAPK3	Mitogen-Activated Protein Kinase 3	Protein Coding	60	GC16M030125
29	IGF1	Insulin Like Growth Factor 1	Protein Coding	59	GC12M102395
30	FOS	Fos Proto-Oncogene, AP-1 Transcription Factor Subunit	Protein Coding	61	GC14P075278
31	CCL2	C-C Motif Chemokine Ligand 2	Protein Coding	57	GC17P034255
32	NOX4	NADPH Oxidase 4	Protein Coding	51	GC11M089324
33	UBC	Ubiquitin C	Protein Coding	51	GC12M124911
34	HIST4H4	Histone Cluster 4 H4	Protein Coding	49	GC12M014767
35	TXN	Thioredoxin	Protein Coding	55	GC09M110243
36	CDKN2C	Cyclin Dependent Kinase Inhibitor 2C	Protein Coding	55	GC01P050960
37	CEBPB	CCAAT/Enhancer Binding Protein Beta	Protein Coding	54	GC20P050190
38	IL1B	Interleukin 1 Beta	Protein Coding	58	GC02M112829
39	RPS27A	Ribosomal Protein S27a	Protein Coding	53	GC02P055231
40	H2AFZ	H2A Histone Family Member Z	Protein Coding	52	GC04M099948
41	CXCL1	C-X-C Motif Chemokine Ligand 1	Protein Coding	51	GC04P073869
42	CDKN2D	Cyclin Dependent Kinase Inhibitor 2D	Protein Coding	52	GC19M010566
43	CCNA1	Cyclin A1	Protein Coding	52	GC13P036431
44	IFNG	Interferon Gamma	Protein Coding	58	GC12M068064
45	PPARG	Peroxisome Proliferator Activated Receptor Gamma	Protein Coding	64	GC03P012328
46	H3F3A	H3 Histone Family Member 3A	Protein Coding	52	GC01P226061
47	KDM6B	Lysine Demethylase 6B	Protein Coding	48	GC17P007834
48	UBB	Ubiquitin B	Protein Coding	53	GC17P016301
49	UBA52	Ubiquitin A-52 Residue Ribosomal Protein Fusion Product 1	Protein Coding	50	GC19P018563
50	HSF1	Heat Shock Transcription Factor 1	Protein Coding	54	GC08P144291
51	HIST1H2A	Histone Cluster 1 H2A Family Member B	Protein Coding	46	GC06M026033
52	HIST1H2A	Histone Cluster 1 H2A Family Member C	Protein Coding	44	GC06P026845
53	HIST1H2A	Histone Cluster 1 H2A Family Member E	Protein Coding	44	GC06P026859
54	HIST2H2A	Histone Cluster 2 H2A Family Member A3	Protein Coding	43	GC01M149843
55	HIST2H2A	Histone Cluster 2 H2A Family Member C	Protein Coding	48	GC01P149886
56	HIST2H2B	Histone Cluster 2 H2B Family Member E	Protein Coding	48	GC01M149884
57	HIST1H2B	Histone Cluster 1 H2B Family Member L	Protein Coding	44	GC06M027808
58	HIST1H2B	Histone Cluster 1 H2B Family Member D	Protein Coding	44	GC06P026813
59	HIST1H2A	Histone Cluster 1 H2A Family Member J	Protein Coding	43	GC06M027816
60	HIST1H2B	Histone Cluster 1 H2B Family Member K	Protein Coding	42	GC06M027138
61	HIST1H2B	Histone Cluster 1 H2B Family Member O	Protein Coding	42	GC06P027895
62	HIST1H2A	Histone Cluster 1 H2A Family Member D	Protein Coding	42	GC06M026198
63	HIST1H4E	Histone Cluster 1 H4 Family Member E	Protein Coding	41	GC06P026204
64	HIST1H2B	Histone Cluster 1 H2B Family Member N	Protein Coding	41	GC06P027844
65	HIST2H2A	Histone Cluster 2 H2A Family Member A4	Protein Coding	34	GC01P149855
66	H2AFV	H2A Histone Family Member V	Protein Coding	46	GC07M044828
67	HIST1H4C	Histone Cluster 1 H4 Family Member C	Protein Coding	46	GC06P026815
68	HIST1H4I	Histone Cluster 1 H4 Family Member I	Protein Coding	45	GC06P027138
69	HIST1H4J	Histone Cluster 1 H4 Family Member J	Protein Coding	45	GC06P027824
70	HIST1H2B	Histone Cluster 1 H2B Family Member A	Protein Coding	45	GC06P025726
71	HIST1H4H	Histone Cluster 1 H4 Family Member H	Protein Coding	44	GC06M026589
72	HIST1H4F	Histone Cluster 1 H4 Family Member F	Protein Coding	44	GC06P026240
73	HIST1H2B	Histone Cluster 1 H2B Family Member C	Protein Coding	44	GC06M026583
74	HIST1H2B	Histone Cluster 1 H2B Family Member J	Protein Coding	44	GC06M027125
75	HIST1H4A	Histone Cluster 1 H4 Family Member A	Protein Coding	44	GC06P026021
76	HIST1H4B	Histone Cluster 1 H4 Family Member B	Protein Coding	44	GC06M026027
77	HIST1H2B	Histone Cluster 1 H2B Family Member H	Protein Coding	44	GC06P026251
78	H2AFJ	H2A Histone Family Member J	Protein Coding	43	GC12P014782
79	HIST1H2B	Histone Cluster 1 H2B Family Member I	Protein Coding	43	GC06P026890
80	HIST1H4K	Histone Cluster 1 H4 Family Member K	Protein Coding	43	GC06M027850
81	HIST1H4D	Histone Cluster 1 H4 Family Member D	Protein Coding	42	GC06M026188
82	HIST1H2B	Histone Cluster 1 H2B Family Member B	Protein Coding	42	GC06M026043
83	HIST1H4L	Histone Cluster 1 H4 Family Member L	Protein Coding	42	GC06M028074
84	HIST1H2B	Histone Cluster 1 H2B Family Member E	Protein Coding	42	GC06P026814
85	HIST3H2B	Histone Cluster 3 H2B Family Member B	Protein Coding	41	GC01P228458
86	HIST1H2B	Histone Cluster 1 H2B Family Member M	Protein Coding	41	GC06P027815
87	HIST1H2B	Histone Cluster 1 H2B Family Member F	Protein Coding	41	GC06P026199
88	HIST2H4A	Histone Cluster 2 H4 Family Member A	Protein Coding	40	GC01P149804
89	HIST1H2B	Histone Cluster 1 H2B Family Member G	Protein Coding	39	GC06M026551
90	HIST2H4B	Histone Cluster 2 H4 Family Member B	Protein Coding	36	GC01M149854
91	H2AFB1	H2A Histone Family Member B1	Protein Coding	34	GC0XP154884
92	LOC10272	Histone H2B Type F-S-Like	Protein Coding	5	GC21M005972
93	VEGFA	Vascular Endothelial Growth Factor A	Protein Coding	61	GC06P043737
94	KL	Klotho	Protein Coding	55	GC13P033016
95	H3F3B	H3 Histone Family Member 3B	Protein Coding	49	GC17M075776
96	ARG2	Arginase 2	Protein Coding	55	GC14P067619
97	FZR1	Fizzy And Cell Division Cycle 20 Related 1	Protein Coding	49	GC19P003506
98	RHOA	Ras Homolog Family Member A	Protein Coding	56	GC03M049397
99	FAS	Fas Cell Surface Death Receptor	Protein Coding	61	GC10P088969
100	KIR2DL4	Killer Cell Immunoglobulin Like Receptor, Two Ig Domains And Lon	Protein Coding	48	GC19P054994
101	CNOT6	CCR4-NOT Transcription Complex Subunit 6	Protein Coding	48	GC05P180494
102	HIST1H3B	Histone Cluster 1 H3 Family Member B	Protein Coding	47	GC06M026031
103	HIST1H3H	Histone Cluster 1 H3 Family Member H	Protein Coding	44	GC06P027810
104	HIST1H3A	Histone Cluster 1 H3 Family Member A	Protein Coding	44	GC06P026020
105	HIST1H3C	Histone Cluster 1 H3 Family Member C	Protein Coding	43	GC06P026062
106	HIST1H3J	Histone Cluster 1 H3 Family Member J	Protein Coding	43	GC06M028077
107	HIST1H3I	Histone Cluster 1 H3 Family Member I	Protein Coding	43	GC06M028073
108	HIST2H3C	Histone Cluster 2 H3 Family Member C	Protein Coding	42	GC01M149839
109	HIST1H3F	Histone Cluster 1 H3 Family Member F	Protein Coding	42	GC06M026250
110	HIST1H3G	Histone Cluster 1 H3 Family Member G	Protein Coding	42	GC06M026269
111	HIST1H3E	Histone Cluster 1 H3 Family Member E	Protein Coding	41	GC06P026889
112	HIST1H3D	Histone Cluster 1 H3 Family Member D	Protein Coding	41	GC06M026582
113	HIST2H3D	Histone Cluster 2 H3 Family Member D	Protein Coding	39	GC01M149814
114	HIST2H3A	Histone Cluster 2 H3 Family Member A	Protein Coding	38	GC01P149857
115	MIR4738	MicroRNA 4738	RNA Gene	14	GC17M075784
116	IL1R1	Interleukin 1 Receptor Type 1	Protein Coding	56	GC02P102136
117	IRAK1	Interleukin 1 Receptor Associated Kinase 1	Protein Coding	58	GC0XM154010
118	FGF2	Fibroblast Growth Factor 2	Protein Coding	57	GC04P122826
119	EHMT2	Euchromatic Histone Lysine Methyltransferase 2	Protein Coding	53	GC06M031879
120	IL1RAPL2	Interleukin 1 Receptor Accessory Protein Like 2	Protein Coding	46	GC0XP104566
121	MAPKAPK	Mitogen-Activated Protein Kinase-Activated Protein Kinase 2	Protein Coding	56	GC01P206684
122	CDC27	Cell Division Cycle 27	Protein Coding	51	GC17M047117
123	ANAPC7	Anaphase Promoting Complex Subunit 7	Protein Coding	46	GC12M110372
124	ANAPC5	Anaphase Promoting Complex Subunit 5	Protein Coding	46	GC12M121326
125	ANAPC4	Anaphase Promoting Complex Subunit 4	Protein Coding	44	GC04P025379
126	UBE2D1	Ubiquitin Conjugating Enzyme E2 D1	Protein Coding	54	GC10P058334
127	IL2	Interleukin 2	Protein Coding	56	GC04M122451
128	NFE2L2	Nuclear Factor, Erythroid 2 Like 2	Protein Coding	55	GC02M177227
129	UBE2C	Ubiquitin Conjugating Enzyme E2 C	Protein Coding	55	GC20P045812
130	ANAPC11	Anaphase Promoting Complex Subunit 11	Protein Coding	48	GC17P081890
131	ANAPC2	Anaphase Promoting Complex Subunit 2	Protein Coding	48	GC09M137174
132	RPS6KB1	Ribosomal Protein S6 Kinase B1	Protein Coding	59	GC17P059893
133	RPS6KA3	Ribosomal Protein S6 Kinase A3	Protein Coding	63	GC0XM020149
134	RPS6KA1	Ribosomal Protein S6 Kinase A1	Protein Coding	60	GC01P026540
135	MAPK7	Mitogen-Activated Protein Kinase 7	Protein Coding	57	GC17P019379
136	EHMT1	Euchromatic Histone Lysine Methyltransferase 1	Protein Coding	55	GC09P137618
137	RPS6KA2	Ribosomal Protein S6 Kinase A2	Protein Coding	55	GC06M166409
138	UBE2E1	Ubiquitin Conjugating Enzyme E2 E1	Protein Coding	51	GC03P023805
139	CDC16	Cell Division Cycle 16	Protein Coding	48	GC13P114234
140	CDC23	Cell Division Cycle 23	Protein Coding	48	GC05M138198
141	ANAPC10	Anaphase Promoting Complex Subunit 10	Protein Coding	47	GC04M144831
142	ANAPC1	Anaphase Promoting Complex Subunit 1	Protein Coding	46	GC02M111768
143	ANAPC16	Anaphase Promoting Complex Subunit 16	Protein Coding	42	GC10P072222
144	CDC26	Cell Division Cycle 26	Protein Coding	42	GC09M113255
145	ANAPC15	Anaphase Promoting Complex Subunit 15	Protein Coding	38	GC11M072106
146	MIR146A	MicroRNA 146a	RNA Gene	21	GC05P160485
147	ILF3	Interleukin Enhancer Binding Factor 3	Protein Coding	48	GC19P010625
148	MMP3	Matrix Metallopeptidase 3	Protein Coding	61	GC11M102835
149	KMT2A	Lysine Methyltransferase 2A	Protein Coding	51	GC11P118436
150	BHLHE40	Basic Helix-Loop-Helix Family Member E40	Protein Coding	48	GC03P004997
151	CD40LG	CD40 Ligand	Protein Coding	57	GC0XP136649
152	ICAM1	Intercellular Adhesion Molecule 1	Protein Coding	60	GC19P010270
153	GATA4	GATA Binding Protein 4	Protein Coding	59	GC08P011676
154	XDH	Xanthine Dehydrogenase	Protein Coding	59	GC02M031294
155	VCAM1	Vascular Cell Adhesion Molecule 1	Protein Coding	57	GC01P100719
156	CXCR2	C-X-C Motif Chemokine Receptor 2	Protein Coding	58	GC02P218125
157	MIR335	MicroRNA 335	RNA Gene	16	GC07P130496
158	G6PD	Glucose-6-Phosphate Dehydrogenase	Protein Coding	59	GC0XM154531
159	ITGB3	Integrin Subunit Beta 3	Protein Coding	59	GC17P047254
160	CDH1	Cadherin 1	Protein Coding	62	GC16P068737
161	MIR24-1	MicroRNA 24-1	RNA Gene	15	GC09P095086
162	HMGB2	High Mobility Group Box 2	Protein Coding	51	GC04M173331
163	CRP	C-Reactive Protein	Protein Coding	56	GC01M159682
164	TLR4	Toll Like Receptor 4	Protein Coding	62	GC09P117704
165	PRKD1	Protein Kinase D1	Protein Coding	56	GC14M029576
166	SPP1	Secreted Phosphoprotein 1	Protein Coding	55	GC04P087975
167	PRKCH	Protein Kinase C Eta	Protein Coding	59	GC14P061187
168	CCR2	C-C Motif Chemokine Receptor 2	Protein Coding	53	GC03P046356
169	TRIM28	Tripartite Motif Containing 28	Protein Coding	51	GC19P058544
170	BRD4	Bromodomain Containing 4	Protein Coding	52	GC19M015236
171	PDGFRB	Platelet Derived Growth Factor Receptor Beta	Protein Coding	66	GC05M150113
172	USP28	Ubiquitin Specific Peptidase 28	Protein Coding	48	GC11M113797
173	MIR15B	MicroRNA 15b	RNA Gene	16	GC03P160404
174	SIRT4	Sirtuin 4	Protein Coding	47	GC12P120291
175	HLA-B	Major Histocompatibility Complex, Class I, B	Protein Coding	55	GC06M031277
176	CEBPG	CCAAT/Enhancer Binding Protein Gamma	Protein Coding	46	GC19P033373
177	PTGS2	Prostaglandin-Endoperoxide Synthase 2	Protein Coding	59	GC01M186640
178	MIR29C	MicroRNA 29c	RNA Gene	17	GC01M207802
179	ADAM17	ADAM Metallopeptidase Domain 17	Protein Coding	61	GC02M009580
180	SLC7A11	Solute Carrier Family 7 Member 11	Protein Coding	53	GC04M138164
181	SELE	Selectin E	Protein Coding	55	GC01M169722
182	IL2RA	Interleukin 2 Receptor Subunit Alpha	Protein Coding	63	GC10M006010
183	CFH	Complement Factor H	Protein Coding	56	GC01P196621
184	A2M	Alpha-2-Macroglobulin	Protein Coding	55	GC12M009067
185	TFE3	Transcription Factor Binding To IGHM Enhancer 3	Protein Coding	52	GC0XM049028
186	ASPSCR1	ASPSCR1, UBX Domain Containing Tether For SLC2A4	Protein Coding	50	GC17P081976
187	IL1RN	Interleukin 1 Receptor Antagonist	Protein Coding	58	GC02P113241
188	MIR24-2	MicroRNA 24-2	RNA Gene	15	GC19M013899
189	NQO1	NAD(P)H Quinone Dehydrogenase 1	Protein Coding	59	GC16M069706
190	ELANE	Elastase, Neutrophil Expressed	Protein Coding	57	GC19P000854
191	DDX58	DExD/H-Box Helicase 58	Protein Coding	57	GC09M032455
192	ATF4	Activating Transcription Factor 4	Protein Coding	57	GC22P039519
193	NFKBIA	NFKB Inhibitor Alpha	Protein Coding	61	GC14M035401
194	ALOX5	Arachidonate 5-Lipoxygenase	Protein Coding	54	GC10P045338
195	CHUK	Conserved Helix-Loop-Helix Ubiquitous Kinase	Protein Coding	61	GC10M100188
196	MPO	Myeloperoxidase	Protein Coding	61	GC17M058269
197	CSF1	Colony Stimulating Factor 1	Protein Coding	54	GC01P109911
198	NFKB2	Nuclear Factor Kappa B Subunit 2	Protein Coding	63	GC10P102394
199	SAA1	Serum Amyloid A1	Protein Coding	51	GC11P018267
200	CX3CL1	C-X3-C Motif Chemokine Ligand 1	Protein Coding	51	GC16P057372
201	MTHFR	Methylenetetrahydrofolate Reductase	Protein Coding	57	GC01M011845
202	ITGAM	Integrin Subunit Alpha M	Protein Coding	56	GC16P031271
203	IKBKB	Inhibitor Of Nuclear Factor Kappa B Kinase Subunit Beta	Protein Coding	64	GC08P042247
204	IL12B	Interleukin 12B	Protein Coding	53	GC05M159314
205	SELL	Selectin L	Protein Coding	51	GC01M169659
206	CD59	CD59 Molecule (CD59 Blood Group)	Protein Coding	58	GC11M033699
207	CD55	CD55 Molecule (Cromer Blood Group)	Protein Coding	57	GC01P207321
208	CD46	CD46 Molecule	Protein Coding	56	GC01P207752
209	FANCA	Fanconi Anemia Complementation Group A	Protein Coding	57	GC16M089740
210	MIR155	MicroRNA 155	RNA Gene	17	GC21P025573
211	PTHLH	Parathyroid Hormone Like Hormone	Protein Coding	55	GC12M027959
212	CTLA4	Cytotoxic T-Lymphocyte Associated Protein 4	Protein Coding	57	GC02P203867
